# Reactive oxygen species (ROS) scavenging biomaterials for anti-inflammatory diseases: from mechanism to therapy

**DOI:** 10.1186/s13045-023-01512-7

**Published:** 2023-11-30

**Authors:** Jiatong Liu, Xiaoyue Han, Tingyue Zhang, Keyue Tian, Zhaoping Li, Feng Luo

**Affiliations:** 1 State Key Laboratory of Oral Diseases, National Clinical Research Center for Oral Diseases, West China School of Stomatology, Sichuan University, Chengdu, 610041 China; 2 Department of Prosthodontics, West China School of Stomatology, Sichuan University, No. 14, Section 3, Renmin Nanlu, Chengdu, 610041 China

**Keywords:** Reactive oxygen species, Biomaterials, Signaling pathways, Inflammatory disease, Anti-inflammation

## Abstract

Inflammation is a fundamental defensive response to harmful stimuli, but the overactivation of inflammatory responses is associated with most human diseases. Reactive oxygen species (ROS) are a class of chemicals that are generated after the incomplete reduction of molecular oxygen. At moderate levels, ROS function as critical signaling molecules in the modulation of various physiological functions, including inflammatory responses. However, at excessive levels, ROS exert toxic effects and directly oxidize biological macromolecules, such as proteins, nucleic acids and lipids, further exacerbating the development of inflammatory responses and causing various inflammatory diseases. Therefore, designing and manufacturing biomaterials that scavenge ROS has emerged an important approach for restoring ROS homeostasis, limiting inflammatory responses and protecting the host against damage. This review systematically outlines the dynamic balance of ROS production and clearance under physiological conditions. We focus on the mechanisms by which ROS regulate cell signaling proteins and how these cell signaling proteins further affect inflammation. Furthermore, we discuss the use of potential and currently available-biomaterials that scavenge ROS, including agents that were engineered to reduce ROS levels by blocking ROS generation, directly chemically reacting with ROS, or catalytically accelerating ROS clearance, in the treatment of inflammatory diseases. Finally, we evaluate the challenges and prospects for the controlled production and material design of ROS scavenging biomaterials.

## Introduction

Inflammation is a fundamental defensive response that eliminates invading pathogens and foreign bodies to restore homeostasis [1]. However, the overactivation of inflammatory responses inevitably harm the host and causes diseases, such as cancer, sepsis, and autoimmunity. This complex biological process that causes clinical symptoms, including heat, pain, redness, and swelling, is broadly triggered by infection and tissue damage [2]. As a basic pathological process, inflammation is associated with most human diseases. For instance, coronavirus disease 2019 (COVID-19), which has caused substantial disruption to health care systems and economies worldwide, is an inflammatory disease, and pneumonia is its most common complication [3]. Despite considerable efforts, inflammation currently remains one of the most complicated and difficult medical challenges in the world. Thus, inflammatory mediators that regulate the occurrence and development of inflammatory responses have become promising therapeutic targets.

The collect term “reactive oxygen species (ROS)” describes the chemical species that are formed upon incomplete oxygen reduction, which are significant mediators of inflammation [4]. ROS mainly include hydroxyl radicals (·OH), superoxide anions (O_2_^−^), singlet oxygen (^1^O_2_), and hydrogen peroxide (H_2_O_2_) [5]. Studies have shown that excessive ROS are highly reactive and can kill cells in the body by oxidizing cellular components, including proteins, lipids, and nucleic acids, leading to inflammation [6]. Additionally, ROS function as signaling molecules and regulate various types of kinases and transcription factors in the initiation and development of inflammation. It is widely believed that ROS participate in inflammatory responses and promote inflammation in a variety of diseases, including diabetes [7], inflammatory bowel disease (IBD) [8], chronic obstructive pulmonary disease (COPD) [9], and osteoarthritis (OA) [10].

**Fig. 1:**
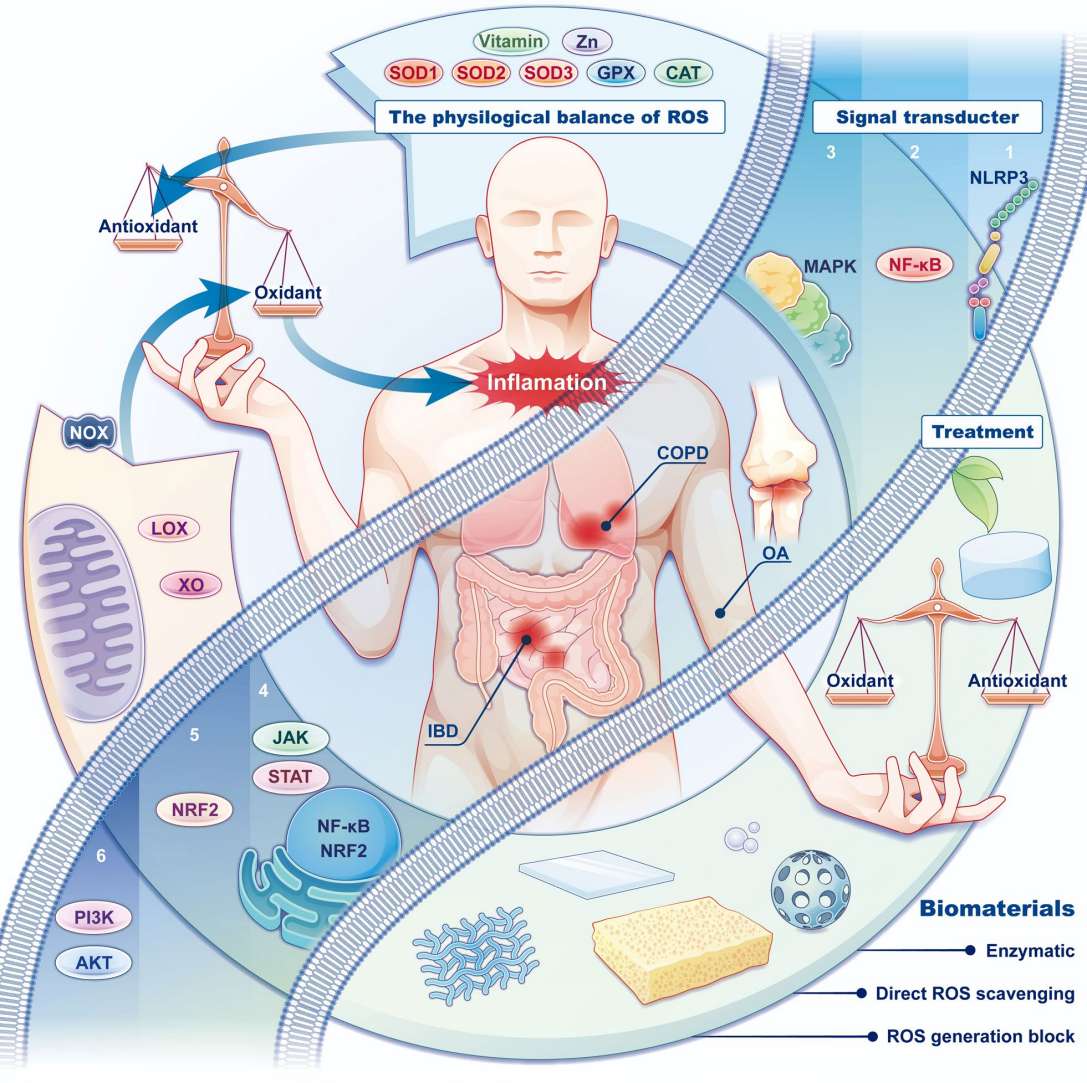
This review systematically outlines the dynamic balance of reactive oxygen species (ROS) production and elimination in physiological states. It further elaborates on the regulatory mechanisms of ROS in inflammatory signaling pathways and discusses the therapeutic potential of ROS scavenging biomaterials for inflammatory diseases, which may help improve the development of anti-inflammatory biomaterials

Based on the close relationship between ROS and inflammation, ROS scavenging biomaterials have been manufactured to limit inflammation and protect the host from damage that is caused by dysregulated inflammatory responses. ROS scavenging biomaterials alleviate inflammation by directly or indirectly balancing the production and elimination of ROS. Traditional anti-inflammatory drugs, such as corticosteroids and nonsteroidal anti-inflammatory drugs, can cause side effects that affect multiple organs, including gastrointestinal and cardiovascular complications and renal failure [11]. Thus, ROS scavenging biomaterials are strong supplements to currently available clinical drugs in the treatment of inflammation [12]. However, current research on ROS scavenging biomaterials lacks a systematic and comprehensive summary of their anti-inflammatory mechanisms. Therefore, it is of great importance to review the molecular mechanisms by which ROS scavenging biomaterials function and classify them according to their scavenging mechanisms; this may guide the further comprehension, design, manufacture, and evaluation of ROS scavenging biomaterials (Fig. 1).

## The physiological balance of ROS

**Fig. 2:**
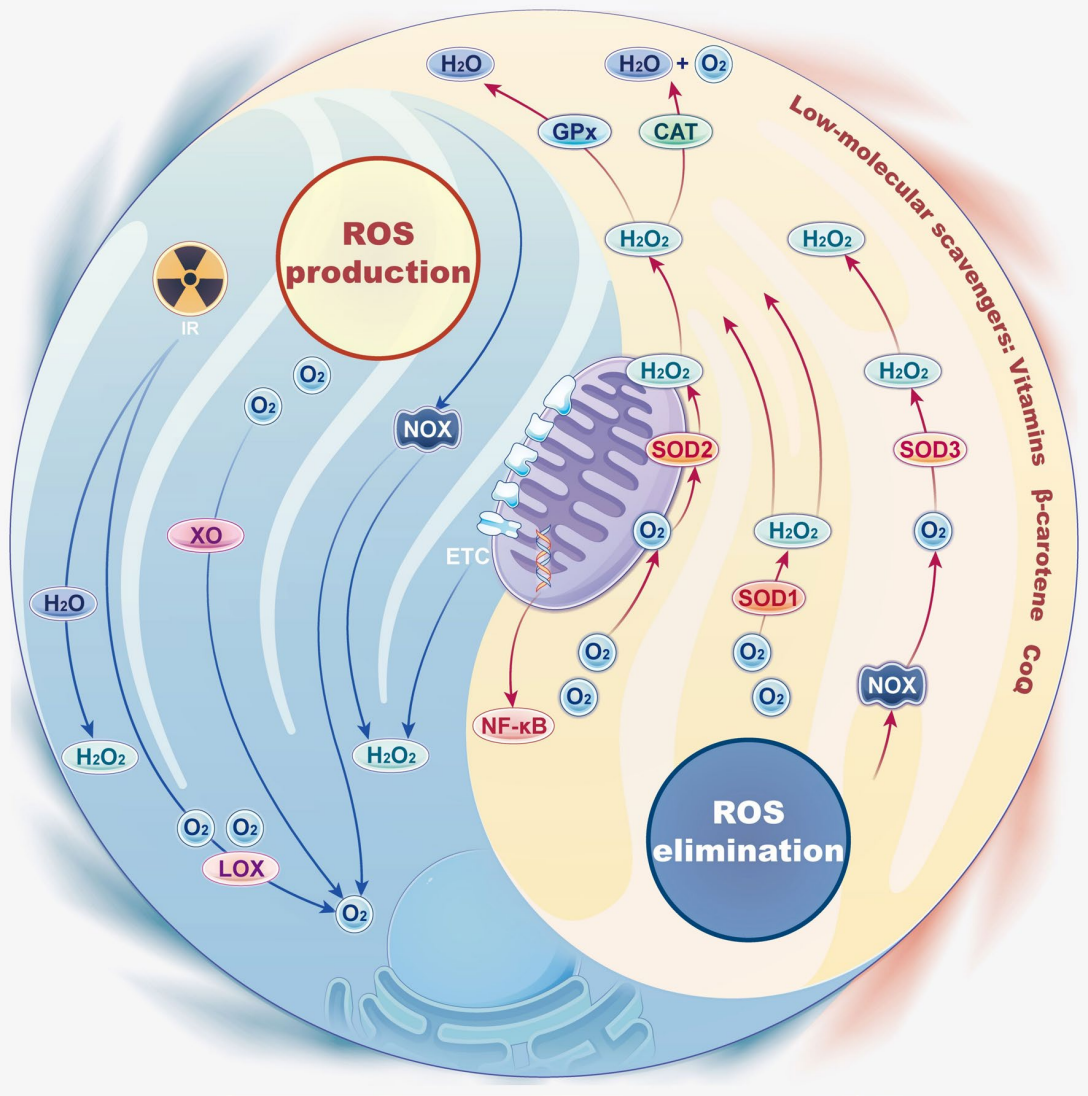
Tai Chi diagram: production and elimination of ROS. ROS are mainly generated from the mitochondrial electron transport chain and as by-products of several cellular enzymes, including NOX, XOR, and LOX. To maintain balance, ROS is controlled with the help of antioxidant networks. The ROS elimination system comprises endogenous antioxidant enzymes and several low-molecular-weight eliminators, including SOD, CAT, GPx, vitamins, β-carotene, coenzyme Q, selenium, and zinc

Under normal physiological conditions, the production and elimination of intracellular ROS are dynamically balanced through different pathways so that ROS can be constantly maintained at relatively low levels [13]. When this balance is stable, ROS play their typical physiological role without causing dysregulated inflammatory responses. However, the overproduction and/or insufficient elimination of ROS may lead to high levels of ROS, resulting in excessive inflammation. Therefore, it is essential to determine the balance of ROS under physiological conditions to understand the working mechanisms and principles of ROS scavenging biomaterials. Here, we introduce three main mechanisms of ROS production and two main mechanisms of ROS elimination (Fig. 2).

### ROS production

**Fig. 3:**
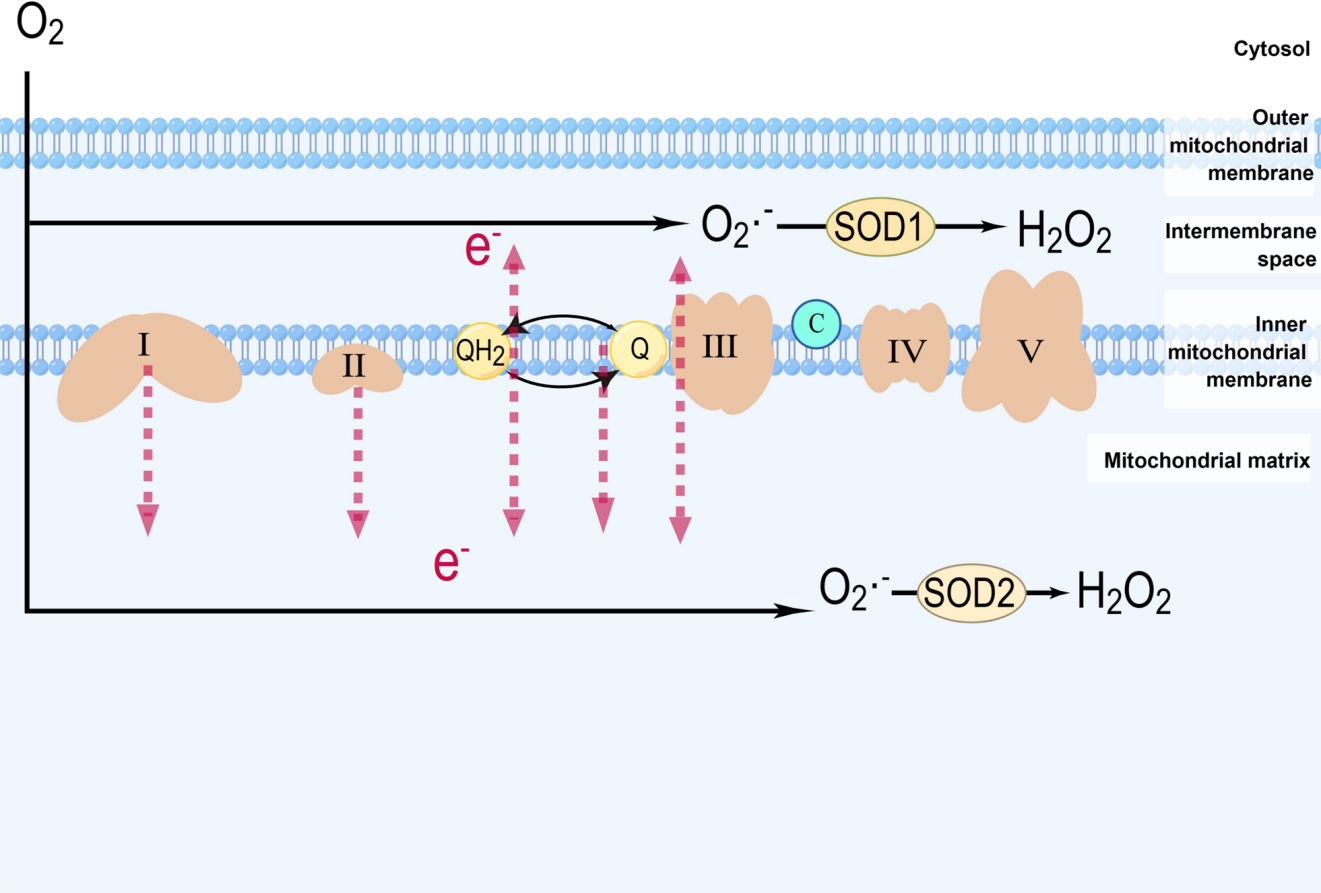
Mitochondrial electron transport chain: oxidative phosphorylation, oxidant production, and measurement methods (created with figdraw). During the cellular oxidation of fuels, electron transfer through the complexes of the ETC is coupled to the genesis of a proton motive force across the mitochondrial inner membrane (MIM). Electrons leak prematurely from complexes I, II, and III to mediate the one-electron reduction of oxygen to O_2_·^−^, which then is dismutated to H_2_O_2_

The majority of ROS originate from the mitochondrial electron transport chain (METC). During the cellular oxidation of fuels, electrons that are transferred through the METC are coupled to the generation of a force that moves protons across the mitochondrial inner membrane (MIM). During this process, most electrons can be safely transferred from donor to acceptor molecules in various redox pathways. However, electrons may prematurely leak from complexes I, II, and III to mediate the one-electron reduction of oxygen to O_2_·^−^, which is then dismutated to H_2_O_2_ (Fig. 3) [14–16]. When the permeability of the mitochondrial membrane increases, ROS that are produced by the METC in mitochondria can be released into the cytosol and induce inflammation via signal transduction or toxic destruction of biological macromolecules [17].

In addition, ROS are also produced as by-products of several cellular enzymes, such as nicotinamide adenine dinucleotide phosphate oxidases (NOXs), xanthine oxidoreductase (XOR), and lipoxygenases (LOXs). The NOX family is a family of multiprotein complexes with 7 members, which include NOX-1 to NOX-5 and dual oxidases (DUOX)-1 and DUOX-2. NOXs generate ROS through the NOX catalytic subunit, which catalyzes the transfer of electrons from nicotinamide adenine dinucleotide phosphate (NADPH) to molecular oxygen. Among all the NOXs, DUOX and NOX-4 primarily generate H_2_O_2_, while the others mainly generate superoxide [18, 19]. In addition, NOX-2 produces ROS during respiratory burst. During this process, NOX-2 is assembled in a phagosome during the phagosome maturation stage and accelerates the reaction of NADPH with molecular oxygen to produce NADP^+^, protons, and O_2_·^−^. Because of the acidic environment of phagosomes, O_2_·^−^ is dismutated into H_2_O_2_, which is subsequently converted to other ROS [19, 20]. XOR is a structurally complicated molybdoflavoenzyme that catalyzes the hydroxylation of xanthine to uric acid. This enzyme has two interchangeable forms in mammals: xanthine dehydrogenase (XDH) and xanthine oxidase (XO). It is believed that XDH is the dominant form that is present in healthy tissues, and it is characterized by NAD^+^ as an electron acceptor. In contrast, XO can produce ROS using O_2_ as a terminal electron acceptor [19]. LOXs are a group of dioxygenase enzymes that catalyze the oxygenation of arachidonic acid (AA) and polyunsaturated fatty acids (PUFAs) to produce hydroperoxyl derivatives [21]. LOXs are designated 5-, 8-, 12-, and 15-LOX, depending on the site where oxygen is inserted in AA. During LOX-catalyzed metabolic processes, ROS are produced as unstable by-products of AA hydroperoxide [22]. In addition, leukotrienes produced by 5-, 12-, and 15-LOX induce NADPH oxides to activate ROS production [23].

Ionizing radiation (IR) is another source of ROS. In general, the production of ROS upon exposure to IR results from water radiolysis. Specifically, low linear energy transfer (LET) IRs, including γ-rays and X-rays, induce the excitation and ionization of water molecules, leading to the production of ROS (mainly free radicals). Furthermore, reactive radicals can also react with other water and oxygen molecules and thus produce more reactive radicals through indirect effects [24].

### ROS elimination

To maintain homeostasis, living organisms aim to control highly reactive ROS via antioxidant networks [15]. The antioxidant system of an organism is defined as an oxidative defense system. This defense system consists of substances in lower concentrations than oxidizable substrates and can delay or prevent oxidation in the body. It is worth noting that the antioxidant defense system should not significantly decrease ROS levels but rather permit sufficient ROS levels to be maintained so they can perform their proper functions. The complicated ROS elimination system comprises endogenous antioxidant enzymes, mainly including superoxide dismutase (SOD), catalase (CAT), and glutathione peroxidase (GPx), as well as low-molecular-weight scavengers, including vitamins, β-carotene, coenzyme Q, selenium and zinc.

Endogenous antioxidant enzymes effectively eliminate ROS through catalysis to prevent inflammation. SODs found in human are divided into cytosolic CuZn-SOD (SOD1), mitochondrial Mn-SOD (SOD2), and extracellular (SOD3) SOD enzymes. SODs seem to be the first line of defense in ROS elimination. They can be rapidly activated under some circumstances and catalyze superoxide into molecular oxygen and H_2_O_2_. This process converts highly reactive ROS into milder species [25]. In addition, CAT neutralizes H_2_O_2_ by decomposing H_2_O_2_ from various sources into molecular oxygen and water [17]. GPxs exert their antioxidant effect mainly by using glutathione (GSH) as a reductant to accelerate the reaction of H_2_O_2_ into water via catalysis [26].

Low-molecular-weight ROS scavengers can activate antioxidant enzymes or terminate oxidative chain reactions [17]. Vitamin D, for example, counteracts the activity of NOX and increases the activity of antioxidant enzymes to accelerate ROS elimination [27]. Vitamin E, as a peroxyl radical scavenger that stops chain reactions, donates its phenolic hydrogen to peroxyl radicals and thus forms tocopheroxyl radicals, which are inactive, thus stopping oxidative chain reactions [28]. In addition, zinc inhibits NOX-2 from exerting its antioxidant effect [29]. However, there are also studies showing that the accumulation of zinc in mitochondria can increase mitochondrial ROS (mtROS) levels and subsequently activate the downstream signaling molecule nuclear transcription factor-kappa B (NF-κB) [30]. In addition to the vitamins and minerals mentioned above, many metabolites, including uric acid, bilirubin, and melatonin, also exhibit antioxidative abilities [17].

Regulating ROS production and elimination in the body to achieve homeostasis is a potential mechanism for achieving anti-inflammatory effects. Many biomaterials have been designed and fabricated based on physiological mechanisms to regulate the levels of ROS. However, the physiological regulatory mechanisms are still poorly understood, limiting the ability to construct a systematic network. We still need to carefully elucidate these mechanisms to prevent additional disruption and disorders in the body.

## ROS signal transduction in inflammation

**Fig. 4:**
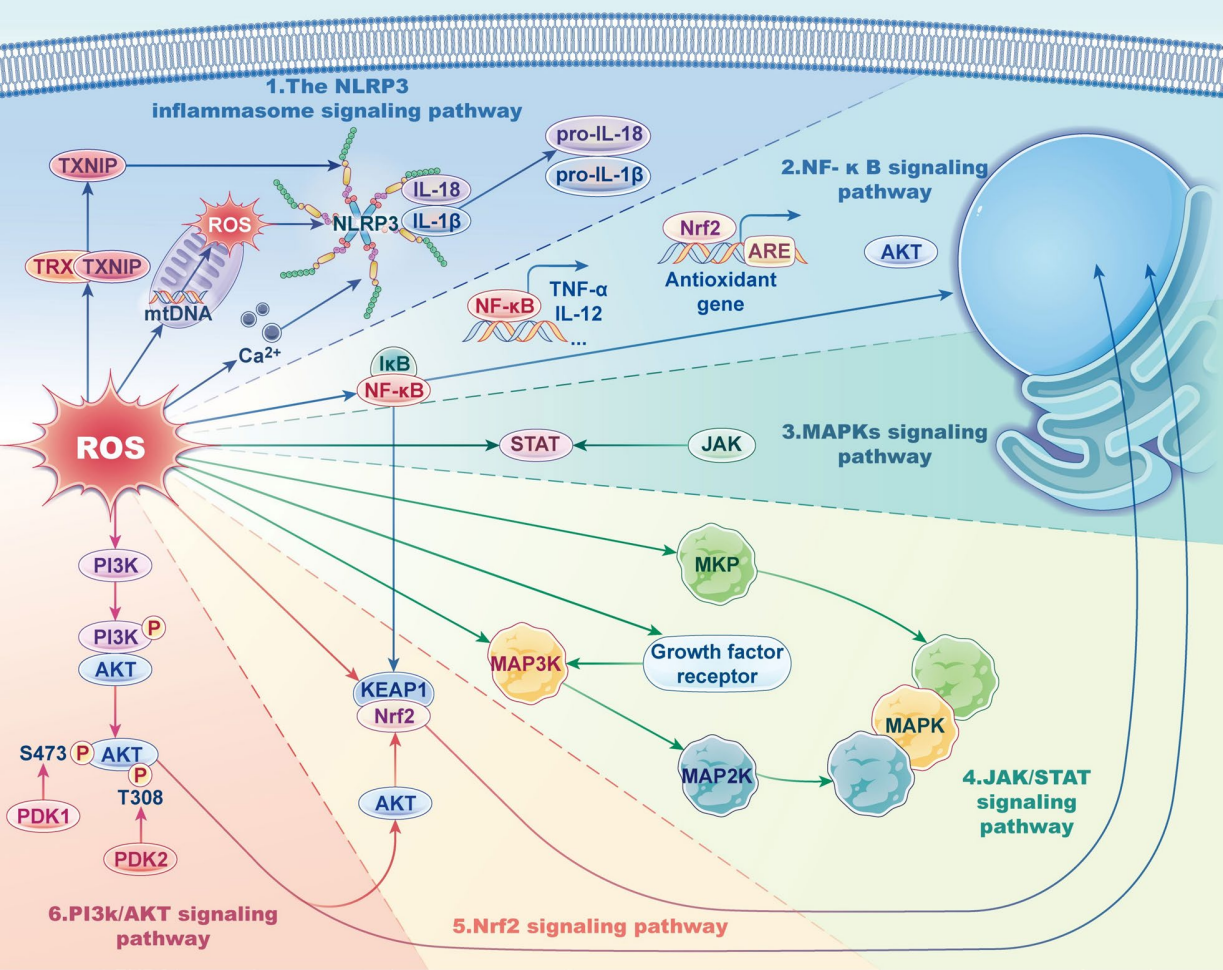
Signal transduction of ROS in inflammation. ROS plays a central role as the second messenger in modifying a variety of signaling molecules to regulate inflammation. They affect multiple inflammatory signaling pathways, including the NLRP3 inflammasome signaling pathway, the NF-κB signaling pathway, the MAPKs signaling pathway, the JAK/STAT signaling pathway, the Nrf2 signaling pathway, and the PI3K/AKT signaling pathway

Classically, ROS are considered lethal defense molecules that are released by neutrophils to destroy exogenous pathogens that invade the body. Different levels of ROS cause different effects. In basic life activities, ROS at the physiological level is in a state of equilibrium and do not damage cells. When the ROS homeostasis are disrupted, superabundant ROS can also damage biomacromolecules, including proteins, lipids, and nucleic acids, in this process, consequently leading to various inflammatory diseases. Nevertheless, an increasing amount of evidence suggests that in contrast to the destruction caused by high levels of ROS, at moderate levels, ROS play central roles as second messengers in modifying a variety of signaling molecules to regulate inflammation [6]. Under pathological conditions, ROS in cells may transmit redox signals through the reversible oxidation of signaling molecules, resulting in permanent changes in inflammatory gene expression [9]. Studies have shown that ROS affect multiple inflammatory signaling pathways, including the nod-like receptor family pyrin domain-containing 3 (NLRP3) inflammasome signaling pathway, NF-κB signaling pathway, and MAPK signaling pathway (Fig. 4). Therefore, a systematic summary of the mechanisms by which ROS act on cell signaling proteins and how these cell signaling proteins further affect inflammation is crucial to thoroughly understand the roles of ROS in inflammation (Table 1).

**Table 1:** ROS signaling pathways

Signaling pathways	ROS targets	Effects	Related diseases	References
The NLRP3 inflammasome signaling pathway	TRX; mtDNA; Ca2 + influx	Production of IL-1β and IL-18; initiation of pyroptosis	Diabetic nephropathy; TRAPS; osteoarthritis; Alzheimer’s disease	[31–47]
NF-κB signaling pathway	IκBα; IKKβ; NIK	M1 macrophage transcription; induction of TNF-α, IL-1β, IL-6, IL-12 and COX-2 gene expression; differentiation of Th1, Th17, and Tfh cells	Inflammatory bowel disease; arthritis; sepsis; gastritis; asthma; atherosclerosis	[6, 9, 48–60]
MAPKs signaling pathway	ASK1; mixed lineage kinase 3; growth factor receptors	M1 macrophage transcription; production of TNF-α, IL-1β, IL-8 and IL-10; decrease in IL-12 and IFN-β production	Asthma; TRAPS; COPD	[6, 9, 37, 61–70]
JAK/STAT signaling pathway	Activation of STAT by DNA methylation	Production of chemokines; differentiation and apoptosis of hematopoietic cells	Rheumatoid arthritis; psoriasis; inflammatory bowel disease	[71–82]
Nrf2 signaling pathway	Keap1; MAPKs; PI3K; NF-κB	Activation of heme oxygenase-1, NADPH dehydrogenase, SOD, and CAT	Acute lung injury; nephritis; osteoarthritis; inflammatory bowel disease	[83–96]
PI3K/AKT signaling pathway	Activation of PI3K; phosphorylation of AKT; inactivation of phosphatase and tensin homology	Expression and activation of inflammatory mediators; recruitment of inflammatory cells; airway remodeling; corticosteroid insensitivity	Chronic inflammatory respiratory diseases	[6, 9, 66, 97–101]

### The NLRP3 inflammasome signaling pathway

Inflammasomes are a group of intracellular multimeric protein complexes that are formed by germline-encoded pattern-recognition receptors (PRRs) [31]; among PRR family members, NLRP3 is the most extensively described. NLRP3 is a tripartite protein that consists of an N-terminal pyrin domain (PYD), a central nucleotide-binding or oligomerization domain (NACHT), and a C-terminal leucine-rich repeats (LRRs) motif [32]. The inflammasome that is formed by NLRP3 is structurally composed of the Nod-like receptor protein NLRP3, the adaptor protein ASC and caspase-1 [33], and this inflammasome plays an important role in destroying exogenous pathogens. Caspase-1 is the effector component of the NLRP3 inflammasome and is activated through proximity-induced autocatalytic activation upon recruitment to the NLRP3 inflammasome. Activated caspase-1 promotes inflammation by cleaving pro-interleukin-1β (pro-IL-1β) and pro-IL-18 into their mature and biologically active forms [31]. In addition to proinflammatory cytokine production, the NLRP3 inflammasome also activates the cleavage of gasdermin D, triggering an inflammatory form of cell death called pyroptosis [34]. Through the effects described above, the NLRP3 inflammasome plays a critical role in inflammation by promoting protective inflammatory reactions. However, dysregulation of the NLRP3 inflammasome results in pathological inflammation [35]. In fact, dysregulation of the NLRP3 inflammasome is related to the pathogenesis of numerous inflammatory disorders, such as diabetic nephropathy (DN) [36], tumor necrosis factor receptor-associated periodic syndrome (TRAPS) [37], OA [38], and Alzheimer’s disease (AD) [39].

Understanding precisely how the NLRP3 inflammasome is activated is crucial for reducing the pathological inflammation that is caused by its dysregulation. However, the mechanism underlying its activation is still poorly understood. The traditional view is that NOX-produced ROS activate the NLRP3 inflammasome [40]. However, increasing numbers of studies have shown that mtROS are the primary mediators of NLRP3 inflammasome activation. An et al. indicated that the *A. baumannii* pathogen pattern-recognition receptor Omp34 activates the NLRP3 inflammasome through mtROS in RAW264.7 cells [41]. More intensively, Nakahira et al. demonstrated that decreased expression of beclin-1 and another autophagy-associated protein, LC3B, might occur upstream of the production of mtROS, ultimately mediating inflammasome activation [42]. Thioredoxin (TRX), which has redox activity, is likely to be the target in NLRP3 inflammasome activation by ROS. Zhou et al. showed that thioredoxin-interacting protein (TXNIP) is released from TRX after the oxidation of TRX by ROS, which enables TXNIP to directly bind to the LRR and NACHT domains of NLRP3 and thus activate the NLRP3 inflammasome [33]. Mitochondrial DNA (mtDNA) is another potential target in NLRP3 inflammasome activation. Shimada et al. showed that mtROS oxidize mtDNA, and its oxidized form binds to NLRP3, activating the NLRP3 inflammasome [43]. In contrast, macrophages that lack mtDNA cannot secrete interleukin-1β (IL-1β) when stimulated by NLRP3 activators. Additionally, it is believed that the ROS-induced Ca^2+^ influx is linked to the activation of the NLRP3 inflammasome. To support the close relationship between Ca^2+^ and the inflammasome, Lee et al. suggested that a mouse Ca^2+^-sensing receptor activates NLRP3 by increasing the intracellular Ca^2+^ concentration, which occurs independently of traditional receptors [44]. In fact, Murakami et al. demonstrated that many NLRP3 activators mobilize Ca^2+^ and that blocking Ca^2+^ signaling can inhibit NLRP3 inflammasome activation [45]. Multiple targets are possible mediators of ROS-induced NLRP3 inflammasome activation. ROS also initiate NLRP3 inflammasome activation through the ROS-dependent transcription factor NF-κB. In turn, NLRP3 inflammasome-mediated activation of inflammatory cells and secretion of IL-1β generate ROS and disrupt the endogenous antioxidant enzymes SOD and CAT, resulting in the accumulation of ROS [46]. In summary, there is likely a positive loop in ROS-mediated NLRP3 inflammasome activation, which suggests that ROS serve not only as triggers but also as effector molecules in activating the NLRP3 inflammasome [46].

However, the mechanism underlying NLRP3 inflammasome activation is still partially controversial. Muñoz-Planillo et al. suggested that ROS production is unnecessary for NLRP3 activation [47]. Instead, the permeation of the cell membrane to K^+^ and Na^+^ is the only mechanism that is needed for the activation of the NLRP3 inflammasome. In addition, Groß et al. revealed a K^+^ efflux-independent mechanism of NLRP3 activation, suggesting that the mobilization of K^+^ is not necessary for NLRP3 inflammasome activation [35]. Although further research is needed to reveal the exact mechanism by which ROS activate the NLRP3 inflammasome, it is clear that ROS and NLRP3 inflammasome activation are related.

### NF-κB signaling pathway

NF-κB is composed of RelA (p65), c-Rel, RelB, p50 (NF-κB1), and p52 (NF-κB2), and this family of transcription factors forms more than twelve different identified heterodimers and homodimers. A conserved Rel homology domain (RHD) is present in all NF-κB subunits, and this domain promotes dimerization and DNA binding [48].

In inactive cells, NF-κB is maintained in the cytoplasm via interaction with a member of the IκB family of inhibitor proteins, such as IκBα. An initiating signal activates the IκB kinase (IKK) complex, phosphorylating IκBα at two N-terminal serine residues. Phosphorylation causes the ubiquitination and proteasomal degradation of IκBα, resulting in the nuclear translocation of NF-κB complexes and the expression of target genes via interaction with high affinity to κB components. This is the most extensively studied canonical pathway of NF-κB activation [9, 48, 49]. In contrast, the noncanonical NF-κB-activating pathway depends on IKKα and activates p52/RelB complexes by triggering the proteolysis of the p52/p100 precursor [6].

A well-acknowledged function of NF-κB is the regulation of inflammation. NF-κB is a core mediator in the induction of proinflammatory gene transcription and the regulation of immune cell functions. In fact, NF-κB is crucial for promoting the transcription of several inflammatory genes, such as those encoding TNF-α, IL-1β, IL-6, IL-12, and cyclooxygenase-2 (COX-2), in M1 macrophages [50]. In addition to mediating the transcription of many proinflammatory genes, NF-κB also regulates inflammatory T cell activation, differentiation, and function. NF-κB facilitates the differentiation of CD4^+^ T cells to T-helper1 (Th1) cells and mediates the production of cytokines such as IL-12, which promote Th1 differentiation. Th1 cells secrete IFN-γ, a critical cytokine that enhances cellular immunity and is involved in inflammatory processes. Furthermore, several NF-κB members have also been demonstrated to facilitate Th17 and T follicular (Tfh) cell responses [50].

NF-κB is chronically activated in various inflammatory disorders, including IBD, arthritis, sepsis, gastritis, asthma, and atherosclerosis [49]. For instance, NF-κB plays an important role in gut homeostasis, and its dysregulation results in an uncontrolled inflammatory state that is commonly observed in IBD. The pathogenesis of IBD is closely related to the expression of various proinflammatory mediators, most of which are produced via the NF-κB signaling pathway. Furthermore, many of the genes that have been shown to be for IBD development can drive NF-κB activation or lead to the dysregulation of NF-κB inhibitory pathways [51].

Currently, increasing numbers of studies have focused on the correlation between ROS and NF-κB. Schreck et al. were the first to indicate (in 1991) that the direct addition of H_2_O_2_ to the culture medium of a subclone of Jurkat cells (Jurkat JR) can activate NF-κB [52]. Later, Chandel et al. demonstrated that mtROS are indispensable for the hypoxic activation of NF-κB [53]. In addition, Lee et al. used a murine model to demonstrate that an antioxidant, L-2-oxothiazolidine-4-carboxylate (OTC), markedly decreases NF-κB translocation into the nucleus and proinflammatory gene transcription. These results proved from another perspective that ROS mediate the activation of NF-κB [54].

**Fig. 5:**
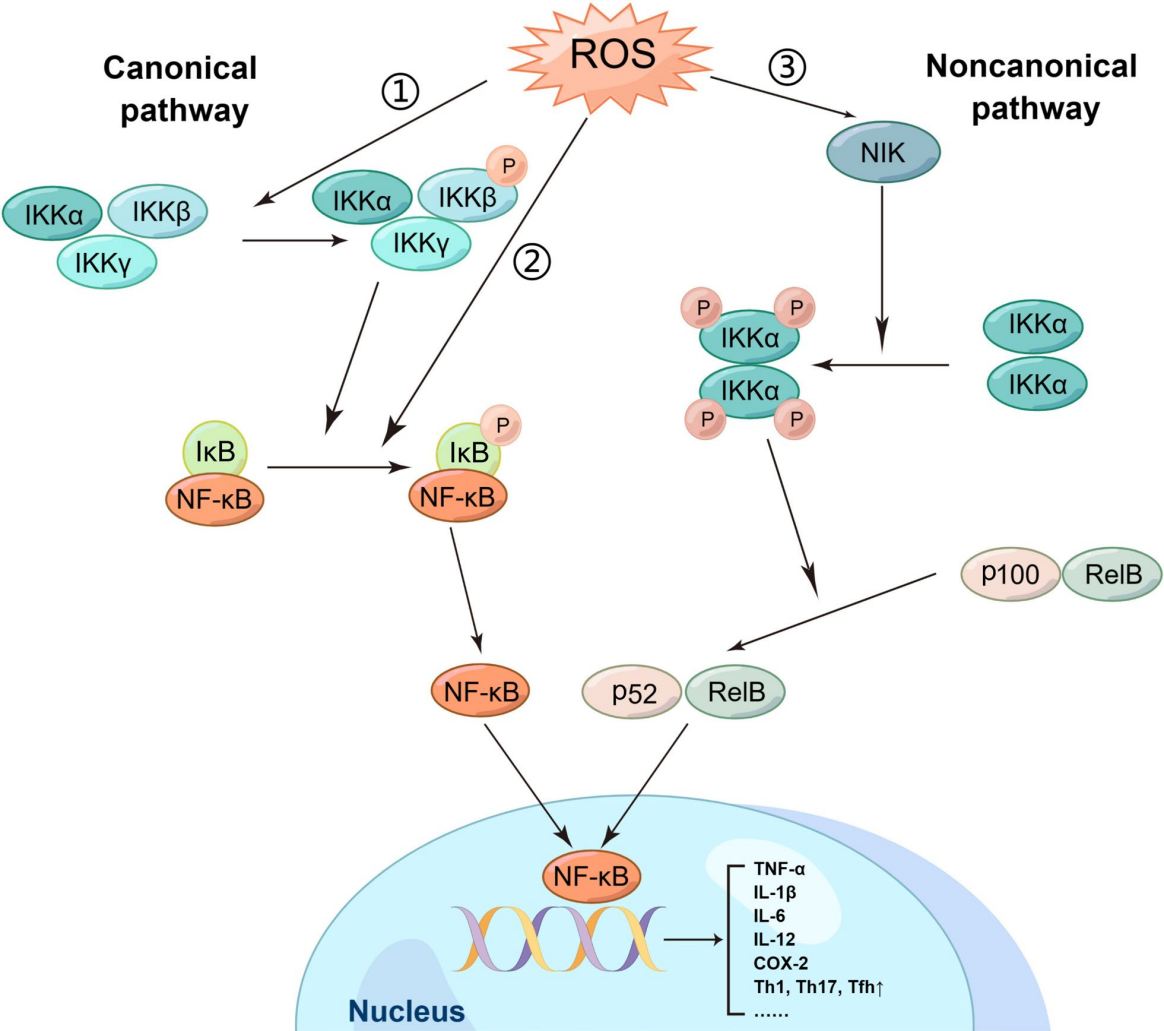
The relationship between ROS and NF-κB signaling pathway (created with figdraw). ROS activate NF-κB through three pathways. Canonical pathway: NF-κB is isolated in the cytoplasm via binding to IκBα. An initiating signal activates the IKK complex and phosphorylating IκBα at two N-terminal serines. The phosphorylation causes the degradation of IκBα, resulting in the nuclear translocation and the activation of target genes. Noncanonical pathway: depends on IKKα and activates p52/RelB complexes by triggering the proteolysis of the p52/p100 precursor

However, how ROS regulate NF-κB remains unclear and controversial (Fig. 5). Takada et al. demonstrated that H_2_O_2_ induces serine phosphorylation in the NF-κB p65 subunit through the Syk-mediated tyrosine phosphorylation of IκBα, leading to its nuclear translocation [55]. Lee et al. showed that treatment with lysophosphatidylcholine (LPC) increases ROS production, which activates NF-κB by triggering the phosphorylation of IκBα in *S. typhimurium*-infected cells [56].

Another possible target in ROS-mediated NF-κB activation is IKKβ. Storz et al. suggested that H_2_O_2_ induces IKKβ activation and NF-κB transcription by activating protein kinase D (PKD) [57]. Song et al. suggested that PM2.5 exposure induces the production of ROS and decreases the expression of miR-331, thus increasing IKK-β expression and continuous NF-κB activation [58]. However, Chen et al. showed that in vitro ablation of IKKβ in fibroblasts results in continuous ROS accumulation, which might indicate that basal IKKβ activity is one of the mechanisms necessary for antioxidants in the body [59].

In addition, NF-κB-inducing kinase (NIK), the upstream kinase in the noncanonical NF-κB signaling pathway, is activated by ROS via the suppression of phosphatases and oxidating cysteine residues. Li et al. demonstrated that H_2_O_2_ mediates NIK activation and the subsequent NIK-mediated phosphorylation of IKKα during the stimulation of NF-κB by IL-1β. However, it is worth noting that NIK is activated by a narrow range of H_2_O_2_ concentrations (1–10 μm). In contrast, higher concentrations suppress NIK activity according to in vitro reconstitution experiments [6, 60].

### MAPK signaling pathways

The mitogen-activated protein kinase (MAPK) cascades, including the extracellular signal-related kinase (ERK), p38 kinase (p38), and c-Jun N-terminal kinase (JNK) pathways, are evolutionarily conserved signaling pathways that mediate several cellular activities, such as inflammation and innate immunity [6]. The activation pathways of these three typical MAP kinases are approximately the same. MAPK signaling cascades are composed of at least three hierarchically sequential kinase compositions: a mitogen-activated kinase kinase kinase (MAP3K), a mitogen-activated kinase (MAP2K), and a MAPK. MAP3Ks activate MAP2Ks by phosphorylation, and activated MAP2Ks phosphorylate and activate MAPKs. Ultimately, activated MAPKs phosphorylate multiple target proteins, mainly MAPK-activated protein kinases (MAPKAPKs), including ribosomal-S6-kinases (RSK1-4), mitogen- and stress-activated kinases (MSK1-2), MAPK-interacting kinases (MNK1-2), and MAPKAPKs (MK) [61].

Together with NF-κB activation, MAPK activation mediates the expression of several genes that collectively regulate inflammation [62]. ERK1/2 activation exerts contradictory effects on inflammation, showing the ability to induce the production of TNF, IL-1β, and IL-10 but inhibiting the production of some proinflammatory mediators, such as IL-12 and interferon-β (IFN-β). The JNK signaling pathway in macrophages promotes inflammatory responses, and this might be related to facilitating the expression of multiple M1 macrophage-specific genes [62]. P38 MAPK consists of four isoforms (α, β, γ, and δ), and p38α and p38β are predominantly involved in inflammatory diseases. Activated p38 phosphorylates histone H3 in the promoters of genes that encode proinflammatory mediators. The phosphorylation ability of p38 also promotes the proximity of NF-κB to its DNA-binding sites. Furthermore, MK2-mediated p38 activation leads to the inactivation of tristetraprolin by phosphorylating its two critical serine residues (Ser52 and Ser178), thus destabilizing several mRNAs that have anti-inflammatory properties. In general, p38 activation results in the increased production of proinflammatory mediators, including IL-1β, IL-8, and tumor necrosis factor-α (TNF-α). Interestingly, while p38α was initially proposed to have mainly proinflammatory properties, considerable evidence has shown that it also plays significant cell type-specific roles in limiting inflammatory responses [63, 64]. These dual effects on inflammation might occur in a cell type-specific manner. In particular, MAPK-mediated inflammation leads to changes in the local inflammatory microenvironment, contributing to tumor invasion [62–64]. Activation of MAPKs induces the expression of multiple inflammatory genes that are related to various inflammatory diseases, such as asthma [9], TRAPS [37], and COPD [63].

Studies have shown that ROS induce the activation of MAPK pathways. Several cellular stimuli that cause ROS production have the ability to activate MAPK pathways in different cell types in parallel [64]. Bulua et al. showed that the ROS scavengers N-acetylcysteine (NAC) and DPI effectively reduce sustained JNK and p38 phosphorylation in TNFR1-heterozygous mutant MEFs and WT MEFs [37]. ROS are associated with the maintenance of MAPK activity and excessive generation of proinflammatory mediators, including IL-6, TNF, IL-8, and IL-10. However, the mechanisms by which ROS activate MAPK pathways remain unclear, principally because of a lack of information regarding the fundamental roles of ROS in activation [64]. MAP3Ks might function critically in redox signaling; among these kinases, ASK1 has been commonly identified as an ROS-responsive kinase. ROS oxidize Trx, a binding protein of ASK1, and its oxidized form dissociates from ASK1. As a result, ASK1 is activated by the phosphorylation of a critical threonine residue in its kinase domain. To support this mechanism, Hsieh et al. suggested that in AML12 hepatocytes, rotenone (ROT)-induced ROS dissociate the Trx-ASK1 complex and consequently activate the p38 MAPK signaling pathway [65]. Pan et al. also demonstrated that scavenging ROS by NAC or CAT decreases ASK1 and p38 MAPK activation [66]. Furthermore, TNF receptor-associated factor 2 (TRAF2) and TRAF6, which are needed to activate ASK1, are recruited to activated ASK1 after ROS stimulation [64]. Regarding another MAP3K, Schroyer et al. suggested that ROS stimulate the phosphorylation of mixed lineage kinase 3 (MLK3) at two serine residues, enhancing MLK3-dependent B-Raf and ERK1/2 activation [67]. Other possible targets of ROS in the activation of MAPK pathways are growth factor receptors, which activate the ERK pathway upstream. The accumulation of ROS induces ligand-independent activation of the EGF receptor (EGFR) and PDGF receptor (PDGFR), subsequently activating the Raf/MEK/ERK signaling pathway [64]. Gonzaga et al. demonstrated a similar process in which acute ethanol intake induces ROS production, PDGFR phosphorylation, and MAPK activation in sequence [68]. The effect of ROS on growth factor receptors might be dose-dependent. Weng et al. showed that mild levels of ROS oxidize PTPs and/or specific Cys residues of EGFR to activate MAPK signaling pathways [69]. In contrast, higher levels of ROS hyperoxidate the Met residue of EGFRT790M and block downstream pathways. Furthermore, MAPK phosphatases (MKPs) dephosphorylate and inactivate MAPKs. Intracellular accumulation of ROS inactivates MKPs through the oxidation of their catabolic cysteine residues. Oxidation results in diminished clearance of MAPKs by MKPs, which promotes the sustained activation of the JNK and p38 pathways. However, this effect depends on the levels of ROS as well. When ROS are present at excessive levels, they activate MKPs and inhibit MAPKs, significantly protecting against the cell death induced by toxic ROS levels [64, 70].

### JAK/STAT signaling pathway

The evolutionarily conserved Janus kinase/signal transducer and activator of transcription (JAK/STAT) signaling pathway is considered a central communication node in regulating inflammation. It consists of ligand‒receptor complexes, namely, JAKs, and STATs. [71]. The JAK/STAT signaling pathway is a direct mechanism by which extracellular factors regulate gene expression. Through transphosphorylation, JAK family members are autoactivated in response to numerous cytokines, such as interleukins, interferons, and hormones. Activated JAKs phosphorylate intracellular receptors domains at specific tyrosine residues, which function as docking sites for STATs. Receptor-localized STATs are subsequently phosphorylated and activated to form dimers, which facilitate their disassociation from the receptors and translocation to the nucleus. In the nucleus, STATs bind to specific adjustment zones in DNA sequences to regulate the transcription of target gene expression. In addition, phosphorylated JAK activates phosphatidylinositol 3-kinase (PI3K), which further activates the PI3K/AKT signaling pathway [71–73].

The JAK/STAT signaling pathway is involved in pathogenetic processes of many inflammatory and autoimmune diseases, such as rheumatoid arthritis, psoriasis, and IBD. JAKs and STATs are extensively employed by various cytokines that are related to the pathogenesis of these diseases to transduce intracellular signals. Moreover, STATs are crucial for regulating the gene transcription of proinflammatory cytokines, which are integral to the development of inflammation. As a result, JAKs and STATs act as indispensable mediators in the development of inflammatory diseases by promoting the production of chemokines and managing the differentiation and apoptosis of hematopoietic cells [74–77]. It should be noted that in regard to the relationship between STATs and inflammation, it must be considered that they can act as receptors for multiple cytokines. Therefore, it is likely that their biological activities are counterregulatory in the context of distinct cytokines. For instance, STAT3 can promote inflammation when activated by IL-6. In contrast, it can inhibit inflammation when activated by IL-10 [77].

While extracellular ligand stimulation directs STAT activation, ROS also modulate the phosphorylation of STAT tyrosine residues [78]. The activation of STAT by ROS may be mediated by DNA methylation [79]. Simon et al. previously suggested that STATs, such as STAT1 and STAT3, are activated in fibroblasts and A-431 carcinoma cells after H_2_O_2_ stimulation, and this activation can be suppressed by antioxidants [80]. Choi et al. showed that pretreatment with the thiol antioxidants GSH and NAC reduces ROS levels and thus attenuates STAT3 activation and reduces inflammation levels in LPS-treated A549 cells [81]. Liu et al. reported that respiratory syncytial virus (RSV)-induced ROS production activates STATs, which occurs independently of tyrosine phosphatases [82]. In general, ROS promote inflammatory responses through the activation of STATs. However, overproduction of ROS and toxic ROS levels may form a redox-sensitive loop, in which ROS inactivate tyrosine phosphatases and further inactivate STAT phosphorylation [78].

The JAK/STAT signaling pathway is a complex pathway, and the underlying mechanisms are still not well understood. This pathway can exert different effects in response to other ligand‒receptor complexes. In addition, the effect of STATs on ROS is also multifaceted, including positive and negative feedback [78]. Therefore, ROS and the JAK/STAT pathway form a complex network relationship, which needs further research and exploration, especially to understand the underlying molecular mechanisms.

### Nrf2 signaling pathway

Nuclear factor erythroid 2-related factor 2 (Nrf2) belongs to the cap ‘n’ collar (CNC) family of transcription factors, which activate the transcription of more than 500 genes, including genes that encode antioxidant enzymes; thus, these transcription factors protect cells from ROS damage and enhance antioxidant activity. Nrf2 contains 605 amino acids and has seven Nrf2-ECH homologous domains (Neh1-7), each of which performs distinct functions in regulating the stability or transcriptional activity of Nrf2. Among these domains, the Neh1 domain is crucial for the function of Nrf2. It forms heterodimer Neh2 domains with small Maf (sMaf) proteins to mediate binding to the cytoplasmic inhibitors of Nrf2 and Keap1 [83]. The Neh2 domain contains a degradation domain and is involved in ubiquitin-dependent degradation [84]. In comparison, the concatenation of the Neh4 and Neh5 domains contributes to the cotransactivation of Nrf2 [85].

The relationship between Nrf2 and ROS is different from that of other pathways. Nrf2 regulates ROS when ROS homeostasis is disrupted, similar to negative feedback effects in organisms. The upstream mechanism that regulates Nrf2 activity is Kelch-like ECH-associated protein 1 (KEAP1). When stimulated by ROS, cysteine residues of KEAP1 are modified, resulting in the phosphorylation of Nrf2, which is translocated to the nucleus to form a heterodimer (Nrf2-MAF) with the Maf protein and the Jun bZip transcription factor. AU-rich elements (IS) in the nucleus can accurately recognize Nrf2-MAF and bind to Nrf2 through its Neh4 and Neh5 domains. Under the guidance of cAMP reaction element binding proteins and transcriptional activators, Nrf2 mediates transcription and thus regulates the expression of genes. Furthermore, a range of antioxidants and phase II enzymes, including heme oxygenase-1 (HO-1), NADPH dehydrogenase, SOD, and CAT, are activated. In this way, harmful substances such as ROS are removed, and cells are protected from oxidative stress, inflammation, and apoptosis. In addition to Keap1, a variety of protein kinases, including MAPKs, protein kinase C (PKC), inositol PI3K and NF-κB, can also induce Nrf2 phosphorylation and participate in Nrf2 transcription [86]. The Nrf2/HO-1 signaling pathway is one of the classical pathways that affects antioxidant enzymes. In addition, the Nrf2/HO-1 signaling pathway significantly reduces the production of mtROS and regulates the integrity of mitochondrial function.

In addition, inflammatory molecules can activate Nrf2 endogenously. For example, 15-deoxyd-d-prostaglandin J2 (15d-PGJ2), one of the end products of the COX-2 pathway, can interact with KEAP1 to activate Nrf2 and thus exert potent anti-inflammatory effects [87]. Notably, the depletion of Nrf2 in mice eliminates the effect of 15d-PGJ2 on attenuating inflammation, suggesting that Nrf2 is crucial for the anti-inflammatory effect of 15d-PGJ2 [88–92].

Currently, some inflammatory diseases can be prevented and/or treated by increasing the levels of Nrf2. In contrast, reducing or knocking out Nrf2 expression increases susceptibility to these diseases. Kong et al. found that LPS-induced TLR and NF-κB signaling was increased in Nrf2-deficient model mice, resulting in increased expression of proinflammatory factors [93]. Furthermore, these authors found that Nrf2 deficiency enhances susceptibility to acute lung injury (ALI) in mice and reverses the attenuation of lung inflammation. In addition to pulmonary inflammation, other inflammatory diseases, including nephritis, OA, and IBD, are associated with Nrf2 deficiency [94–96]. Nrf2 pathway is a signaling pathway that is activated by disrupted ROS homeostasis to scavenge ROS and thus exert anti-inflammatory effects.

### PI3K/AKT signaling pathway

PI3K and its target protein, protein kinase B (PKB), are essential cell signaling molecules. They play significant roles in cell apoptosis and proliferation by influencing the functions of many downstream molecules. PI3K mainly comprises a regulatory subunit (P85) and a catalytic subunit (P110), which are activated by tyrosine kinase receptors. When cell membrane receptors are stimulated by various extracellular factors, including cytokines, growth factors, and hormones, intracellular PI3K is activated. Subsequently, bioactive PI3K catalyzes the transition of phosphatidylinositol 4,5-diphosphate (PIP2) to phosphatidylinositol 3,4,5-triphosphate (PIP3) [97]. This reaction leads to the recruitment and activation of proteins containing the pleckstrin homology (PH) domain, including phosphoinositol-dependent protein kinase (PDK) and the serine/threonine protein kinase AKT (also known as PKB).

There are three closely related subtypes of AKT, and they play significant roles in regulating cell growth, multiplication, survival, and metabolism [98]. AKT is the crucial protein downstream of PI3K and consists of PH, catalytic, and regulatory domains. Loss or mutation of the PH domain may lead to a decrease and inactivation of AKT. Activation of PI3K leads to an interaction between PIP3 and AKT, which causes the translocation of AKT from the cytoplasm to the membrane. Moreover, the conformation of AKT changes, exposing its threonine and serine protein. AKT is activated only when both residues are phosphorylated [99]. Then, activated AKT is transferred from the membrane back to the cytoplasm or nucleus. Activated AKT further targets downstream signaling molecules, including mTOR, Bad, caspase 9, cyclin D1, and NF-κB [100].

ROS can simultaneously activate PI3K to directly amplify its downstream signal and inactivate phosphatase and tensin homology (PTEN). PTEN negatively regulates PIP3 synthesis via cysteine residues in the active oxidizing center and consequently inhibits the activation of AKT [6]. In addition, ROS can facilitate the phosphorylation of PTEN through casein kinase II, which promotes the entry of PTEN into the proteolytic degradation pathway.

The PI3K-AKT pathway is associated with inflammation. Studies have demonstrated that the activation of the PI3K/AKT signaling pathway can inhibit OA and chondrocyte apoptosis in rats. Inhibition of this pathway can result in the opposite effect [101]. Therefore, the PI3K-AKT pathway exerts specific effects via anti-inflammatory mechanisms. However, several studies have shown that this pathway can also promote inflammation. Many constituents of the PI3K pathway play positive roles in the expression of inflammatory cytokines, the recruitment of inflammatory cells, and the function of immune cells [9]. In addition, ROS have been demonstrated to regulate the phosphorylation of AKT [66], which then induces inflammation in various cell types. As a signaling pathway that plays a possible role in the pathogenesis of inflammation, the PI3K pathway is still expected to be a breakthrough in the treatment of inflammation. Therefore, further studies on mechanisms related to PI3K/AKT signaling are needed.

## ROS scavenging biomaterials in the treatment of inflammation

**Fig. 6:**
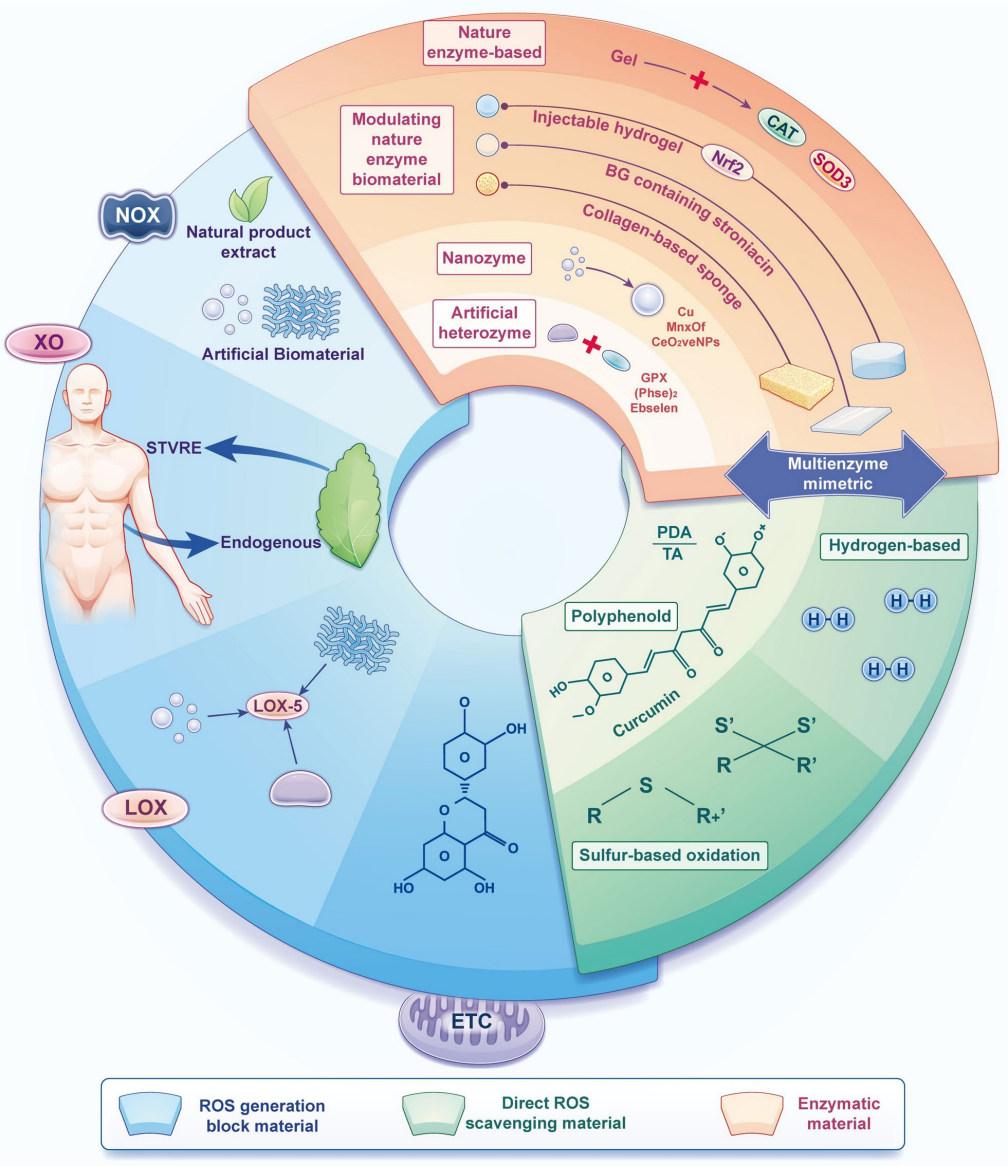
Summary of ROS scavenging materials. Based on their diverse working mechanisms, ROS scavenging biomaterials are classified into three categories. Enzymatic biomaterials: enzymatic biomaterials that mimic enzymes or enhance the action of natural enzymes to accelerate ROS removal through catalysis; direct ROS scavenging biomaterials: biomaterials that react directly with ROS; ROS generation-blocking biomaterials: biomaterials that block ROS sources to reduce ROS production

When ROS-mediated inflammatory diseases occur, the physiological ROS scavenging system cannot protect the body against effects of ROS overproduction. Hence, biomaterials with potent ROS scavenging abilities are considered promising therapeutic agents for inhibiting inflammation [102]. Recently, biomaterials with distinctive ROS scavenging properties have been designed, and these biomaterials have potential activities to overcome the fundamental challenges of treatments that target ROS and inflammation in clinical settings [12]. Based on their diverse working mechanisms, ROS scavenging biomaterials are classified into three categories: enzymatic biomaterials that mimic enzymes or enhance the action of natural enzymes to accelerate ROS elimination through catalysis, biomaterials that directly react with ROS, and biomaterials that block ROS sources to reduce ROS production (Fig. 6). This classification provides insight into the anti-inflammatory therapeutic mechanisms of ROS scavenging biomaterials, and it may guide further understanding, design, manufacture, and evaluation of these materials. This section will summarize the working mechanisms of ROS scavenging biomaterials and the most recent advances in the use of these materials for treating ROS-mediated inflammatory diseases (Table 2).

**Table 2:** Biomaterials for ROS scavenging

Materials			Working mechanisms		Applications	References
Enzymatic biomaterials	Natural enzymes-based biomaterials	CAT-based materials	Decomposition of hydrogen peroxide into molecular oxygen and water		Anti-inflammation wound dressing	[103–105]
		SOD-based materials	Catalysis of superoxide into oxygen and hydrogen peroxide		Chronic wound healing; trauma treatment	[106, 107]
	Modulating natural enzymes biomaterials	Nrf2-activating materials	Activation of Nrf2		Skin inflammation; traumatic brain injury; osteoporotic bone defects	[108–110]
		Collagen-based sponge	Activation of SOD and GPx		Wound healing	[111, 112]
		Bioactive glasses containing Sr	Activation of SOD, CAT, and GPx		Repair and regeneration of wound and bone	[113, 114]
	Nanozymes	Ce-contained nanozymes	SOD- and CAT-mimetic nanozyme activities		Periodontitis; Acute lung injury; Spinal cord injury; Refractory wound healing; Diabetic wound healing Age-related macular degeneration; Premature skin aging and cancer; Sepsis	[117, 118, 121–123]
		Mn-contained nanozymes	SOD- and CAT-mimetic nanozyme activities		Ear inflammation; Gingivitis; Periodontitis	[116, 128–130]
		Cu-contained nanozymes	GPx-, SOD-, and CAT-mimetic nanozyme activities		Acute kidney injury; Acute liver injury; Wound healing	[132, 133]
		Pt-contained nanozymes	GPx-, SOD-, and CAT-mimetic nanozyme activities		Osteoarthritis; UVB-induced inflammation	[134–136]
		Prussian blue-contained nanozymes				[137–139]
		Carbon dots nanozymes				[140, 141]
		Prussian blue-contained nanozymes	POD-, SOD-, and CAT-mimetic nanozyme activities		Osteoarthritis; Hepatic ischemia‒reperfusion injury; Wound healing	[246–248]
		Carbon dots nanozyme	SOD-mimetic nanozyme activities		Ulcerative colitis	[249, 250]
	Artificial selenoenzymes	Diphenyl diselenide	GPx-mimetic activity		Chronic *T. gondii* infection; Ischemia/reperfusion; Amyotrophic lateral sclerosis	[143–145]
		Ebselen	GPx-mimetic activity		Influenza; Glioma; *H. pylori*-induced cell damage	[146–148]
Direct ROS scavenging biomaterials	Polyphenols	Curcumin	Free radical scavenging by H-atom transfer or electron transfer		Neuroprotection; Wound healing; Ischemia‒reperfusion injury; Acute kidney injury; Acute liver injury; Ankle inflammation	[151–162, 251]
		Polydopamine	Free radical scavenging; Chelation of metal ions; SOD-mimetic activity		Periodontitis; Acute kidney injury; inflammatory bowel disease	[163–171]
		Tannic acid	Free radical scavenging; Chelation of metal ions		Wound healing; Inflammatory bowel disease; Liver injury; Ischemic stroke; Cognitive impairment; Skin protection; Bone renovation and regeneration; Osteoarthritis	[173–186]
	H2-based materials	–	Chemical reactions with ROS; Activation of Nrf2; Activation of SOD and GSH; Downregulation of NOX-2		Airway disease; Periodontitis; Psoriasis; Foot inflammation	[187–197]
	Sulfur-based oxidation-responsive biomaterials	Thioether/sulfoxide-contained biomaterials	Redox reactions with ROS		Neuroprotection; Diabetes; Interstitial cystitis; Rheumatoid arthritis	[198, 201–209]
		Thioacetals/thioketals-contained biomaterials	Redox reactions with ROS		Ankle inflammation; Ischemic wound healing; Repair of bone defects	[151, 202]
		Hydrogen sulfide	Redox reactions with ROS		Cardiovascular inflammatory diseases; Neurodegenerative diseases	[210–213]
	Bilirubin-derived biomaterials	–	Free radicals scavenging by H-atom; Bilirubin–biliverdin cycle; Intranuclear regulation concerning Nrf2, HO-1, and antioxidants		Asthma; acute pancreatitis; osteoarthritis; Ulcerative colitis; Cardiac ischemia–Reperfusion injury; Hepatic ischemia–Reperfusion injury; Rosacea; Psoriasis; Pulmonary fibrosis	[218–228]
ROS generation-inhibiting biomaterials	NOX-inhibiting biomaterials	–	Downregulation the production of ROS by inhibiting NOX		Arthritis; Ulcerative colitis; Asthma; Ischemia/reperfusion-Induced brain injury	[229, 230, 232, 252, 253]
	XO-inhibiting biomaterials	–	Downregulation the production of ROS by inhibiting XO		Anti-inflammation; Hyperuricemia	[233, 234]
	LOX-inhibiting biomaterials	–	Downregulation the production of ROS by inhibiting LOX		Peritonitis; Asthma	[236, 237]
	ETC-regulating biomaterials	–	Downregulation the production of ROS by regulating the activities of ETC		Alzheimer’s disease	[239–242, 254]

### Enzymatic biomaterials

#### Natural enzyme-based biomaterials

Natural enzymes have promising applications in biotechnology, biomedicine, and pharmacy. For instance, natural enzymes with broad-spectrum abilities have been proven to be efficient biotherapeutics in the treatment of various inflammatory diseases caused by dysregulation of the ROS scavenging system [12]. Nevertheless, the effective delivery of these natural enzymes in treatment is generally limited by their inherent low stability under working conditions, high sensitivity to fabrication and storage, and short half-lives [103]. To make the use of natural enzymes more efficient, a variety of natural enzyme-based biomaterials have been designed and fabricated.

For instance, Abdel-Mageed et al. prepared a gelatin (Gel)–alginate (Alg) biocompatible hydrogel (Gel–Alg) using calcium chloride as an ionic cross-linker [103]. The Gel-Alg hydrogel could act as an immobilization support to improve CAT stability for practical applications. As a result, the integration of the natural antioxidant CAT into Gel–Alg holds promise as a novel anti-inflammatory wound dressing. Other studies have also pointed out that there are many ways to support CAT, such as glutaraldehyde cross-linked BSA hydrogels and methacrylate gelatin (GelMA) inverse opal scaffolds [104] or nanoporous gold (NPG) [105]. The biocomposites described above have enormous application potential, including in the inhibition of inflammation.

Biomaterials loaded with SOD have also been intensively studied. Zhuang et al. modified γ-PGA with taurine (γ-PGAS) and prepared a SOD-loaded γ-PGAS/γ-PGA hydrogel (SOD-PGAS/PGA-H) through cross-linking. Compared with natural SOD, SOD-PGAS/PGA-H combines the strengths of SOD and γ-PGAS/γ-PGA-H. It has a stronger capability to scavenge ROS and generates a moist microenvironment, promoting chronic wound healing [106]. Similarly, Dong et al. designed a new type of thermosensitive hydrogel, poly(N-isopropyl-acrylamide)/poly(gamma-glutamic acid) (PP), loaded with SOD, which showed the capability to inhibit or reduce ROS generation and improve the treatment of traumatic wounds [107].

#### Modulating natural enzyme biomaterials

At present, the modulation of therapeutic biomaterials is focused on regulating the effects of natural cells and biological macromolecules in the human body to accelerate targeted treatment with either the body’s own systems or natural products. Modulating natural enzyme biomaterials strengthens the physiological enzymatic ROS defense system to clear ROS and reduce inflammation by enhancing the physiological activity of natural ROS scavenging enzymes, including SOD, CAT, and GPx.

Nrf2 is an important target for modulating natural enzyme biomaterials by activating various antioxidant enzymes, including HO-1, NADPH dehydrogenase, SOD, and CAT. Zhang, D. et al. prepared an injectable hydrogel that activates the Nrf2 signaling pathway by combining gallic acid grafted hyaluronic acid (HA) with HA-tyramine (HT) polymer via a dual-enzyme cross-linking method [108]. The hydrogel inhibits neuroinflammation, contributing to the repair of traumatic brain injury (TBI) by decreasing the levels of a variety of proinflammatory cytokines, including TNF-α and IL-6, and increasing the expression of the anti-inflammatory cytokine IL-4. Guided by numerous anti-inflammatory biomaterials that successfully activate Nrf2, Park et al. showed that malonic acid (MA) isolated from *Pinus densiflora* promotes the antioxidant enzymes SOD1 and HO-1 through activation of Nrf2, resulting in a reduction in UVB-induced ROS levels [109]. Consequently, MA reduces the ROS-induced activation of NF-κB, MAPK, and proinflammatory cytokines (IL-6, COX-2, and TNF-α). In addition, Qian, Z.J. et al. demonstrated that two peptides from the seahorse (SHP-1 and SHP-2) activate Nrf2 and notably reduce intracellular ROS levels [110]. Although they are currently not applied in the treatment of inflammatory diseases, MA isolated from *Pinus densiflora* and SHP-1 and SHP-2 isolated from seahorse hydrolysates are biomaterials with highly potential anti-inflammatory functions.

Collagen is rich in glycine, alanine, and glutamic acid, but it contains low levels of tyrosine and phenylalanine. Collagen can potently improve the activities of SOD and GPx in cultured RAW264.7 cells, leading to ROS scavenging and protection against H_2_O_2_-induced inflammation [111]. Aravinthan et al. showed that a collagen-based sponge is an effective material for dressing open wounds that can significantly decrease IL-6 and TNF-α production and increase anti-inflammatory cytokine IL-10 production in wound tissues [112]. In addition, wound tissues treated with the collagen-based sponge exhibit apparent reductions in inflammatory cells, suggesting that this sponge is an emerging material for wound healing.

Furthermore, bioactive glasses (BG) containing strontium are another type of biomaterial with modulated natural enzymes, and it can increase the activities of SOD, CAT, and GPx. BG-sr exerts a unique protective effect on ROS in the body, decreasing the inflammatory response induced by ROS and playing an important role in the repair and regeneration of wounds and bone [113, 114].

#### Nanozymes

With the rapid and notable development of nanotechnologies, it has been discovered that nanoparticles have an intrinsic ability to mimic the catalytic activity of some biological enzymes; these particles are called nanozymes [115]. Many nanozymes have been utilized because of their distinctive ROS scavenging abilities, and they exhibit the potential to overcome the core difficulties of anti-ROS therapy. Overall, nanozymes have numerous advantages, such as enhanced stability, multifunctionality, and tunable activity. At present, various nanostructures that can catalytically eliminate ROS have already been developed [12, 116].

##### Ce-contained nanozymes

**Fig. 7:**
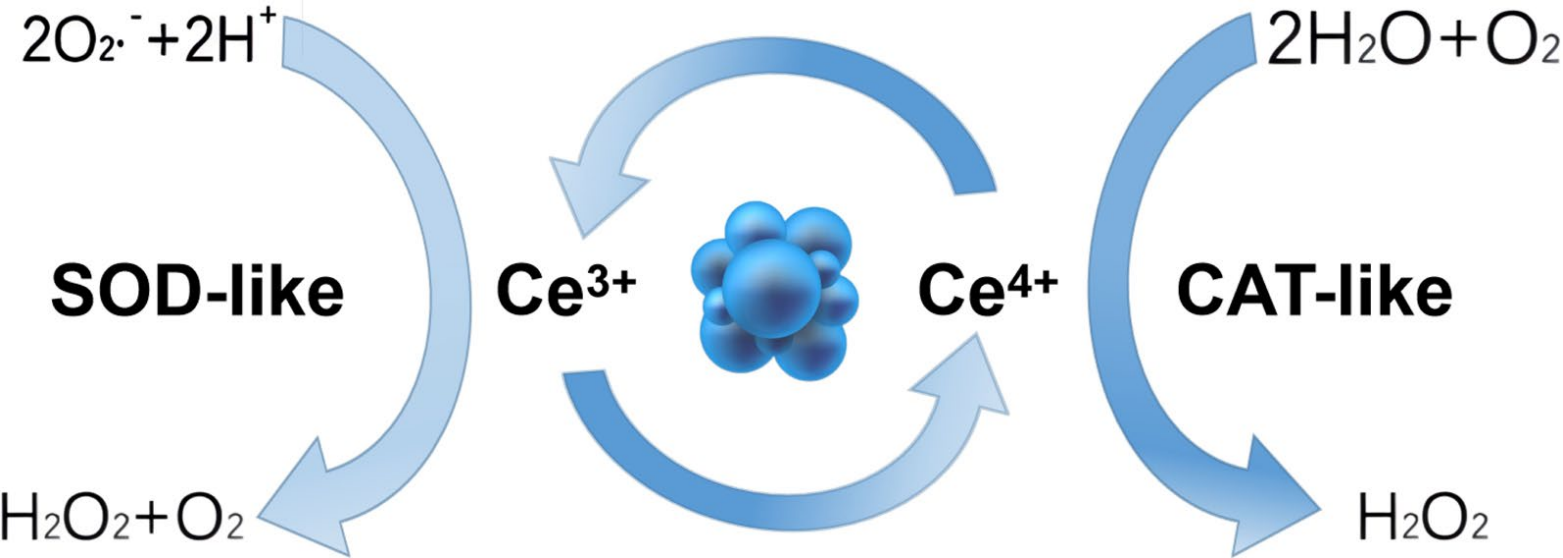
Mechanism of ROS removal by CeO_2_ NPs (created with figdraw). Ce^3+^ on the surface transforms superoxide radicals into oxygen and H_2_O_2_, while Ce^4+^ scavenges H_2_O_2_ and generates oxygen and water, ultimately eliminating ROS. Due to the absorption of hydrogen electrons, Ce^4+^ is converted into the original Ce^3+^. The recyclable changes allow CeO_2_ NPs could be used as SOD mimetics and CAT mimetics as a logical therapeutic agents in treating ROS-induced inflammatory diseases

The biological applications of cerium oxide nanoparticles (CeO_2_ NPs) in scavenging ROS have received extensive attention in recent decades. CeO_2_ NPs are unique due to the convertible surface, which contain both trivalent cerium atoms (Ce^3+^) and tetravalent cerium atoms (Ce^4+^). Ce^3+^ on the surface serves as an analog of SOD, which transforms superoxide radicals into oxygen and H_2_O_2_. In comparison, Ce^4+^ produced by the abovementioned reactions scavenges H_2_O_2_ and generates oxygen and water, ultimately eliminating ROS. Ce^4+^ is converted into the original Ce^3+^ via the absorption of hydrogen electrons. In general, the recyclable changes between Ce^3+^ and Ce^4+^ allow CeO_2_ NPs to be used as SOD and CAT mimetics (Fig. 7). Therefore, they are logical therapeutic agents for treating ROS-induced inflammatory diseases [117].

**Fig. 8:**
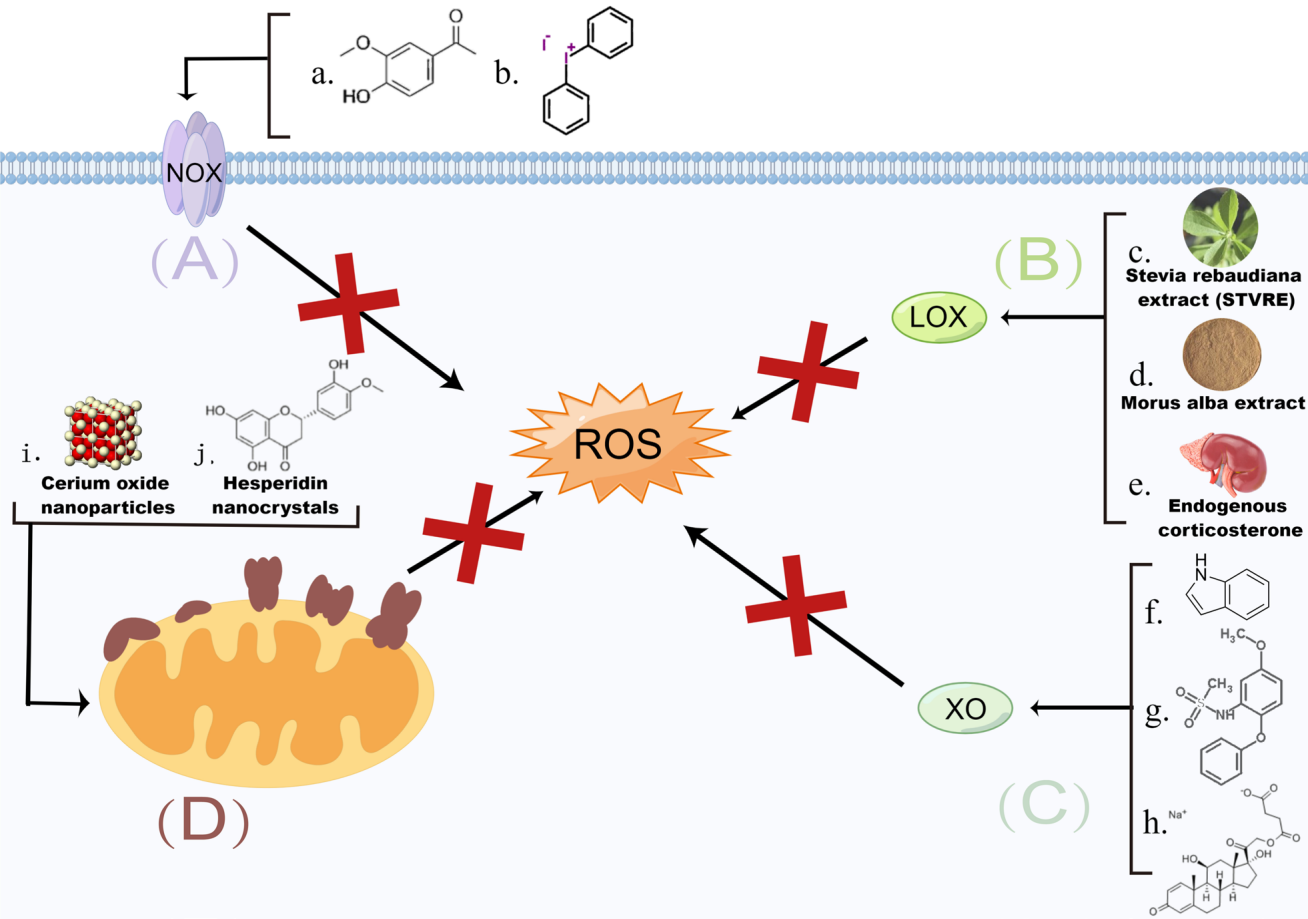
Mechanism of ROS generation-blocking materials (created with figdraw). **A** NOX-inhibited materials. **B** LOX-inhibited materials. **C** XO-inhibited materials. **D** ETC-regulated biomaterials

Yu et al. demonstrated that CeO_2_ NPs scavenge multiple ROS and inhibit the MAPK and NF-κB signaling pathways to decrease proinflammatory mediators [118]. In particular, CeO_2_ NPs can markedly suppress inflammation in rat periodontitis models. Niemiec et al. found that CeO_2_ NPs conjugated to microRNA-146a (CNP-mir146a) and delivered through the trachea increase pulmonary levels of miR146a without causing systemic increases [119]. This indicates that CNP-miR146a shows potential in preventing ALI. The NF-κB signaling pathway and the inflammatory pathways it activates have been shown to be key mediators in the pathogenesis of ALI and acute respiratory distress syndrome (ARDS). CNP-miR146a inhibits the NF-κB signaling pathway and prevents ALI by altering leukocyte recruitment and reducing inflammation and oxidative stress. In addition to ALI, CNP-miR146a can improve diabetic wound healing. Dewberry et al. demonstrated that CeO_2_ NPs act as free radical scavengers, while miR146a inhibits the proinflammatory NF-κB pathway and synergistically regulates oxidative stress and inflammation [120] (Fig. 8).

Huang et al. prepared chitosan-coated CeO_2_ nanocubes (CCNs) through a hydrothermal method. These authors demonstrated that CCN application results in notable wound healing due to their anti-inflammatory effects, which decrease TNF-α and increase IL-10 [121]. CCNs have shown great potential in treating refractory wounds caused by persistent inflammation in oxidative stress-related diseases, including diabetes. Injection of CeO_2_ NPs has also been confirmed to reduce inflammation in the corresponding target sites; thus, these NPs have promising prospects in the treatment of age-related macular degeneration (AMD) [122] and spinal cord injury (SCI) [123].

In addition, Ribeiro and Peloi et al. demonstrated that CeO_2_ NPs can protect L929 fibroblasts from ultraviolet-A radiation (UV-A)- and ultraviolet-B radiation (UVB)-induced damage [124, 125]. Exposure to ultraviolet radiation is a major cause of premature skin aging and cancer, which is mainly caused by the overproduction of ROS. CeO_2_ NPs reestablish oxidation balance by ameliorating ROS levels and enhancing antioxidant enzyme activity, thereby preventing UV-mediated oxidative damage to L929 cells. The team also demonstrated that CeO_2_ NPs mitigate the effects of neutrophil oxidation reactions by reducing cell damage, suggesting that CeO_2_ NPs can potentially be used as radioprotective/therapeutic agents for UV damage [126].

In addition to CeO_2_ NPs, ceria-zirconia nanoparticles (CZ NPs) act as antioxidants and have shown significant performance in the treatment of inflammatory conditions. Soh et al. synthesized 2 nm CZ NPs with a higher Ce/Ce ratio and faster conversion rate [127]. The resulting CZ NPs have significantly improve ROS scavenging performance and thus regulate inflammatory cells at very low doses. Furthermore, CZ NPs have been shown to be effective in reducing mortality and systemic inflammation in two representative sepsis models. These findings suggest that CZ NPs have potential as therapeutic nanomedicines for the treatment of ROS-related inflammatory diseases.

##### Manganese-contained (Mn-contained) nanozymes

Mn-contained nanozymes have shown extensive research prospects due to their excellent performance [128]. Mn-contained nanozymes mainly include MnO, MnO_2_, Mn_2_O_3_, Mn_3_O_4_, and other manganese oxide nanozymes, which have become a current focus of research [129]. In general, Mn-contained nanozymes exhibit SOD- and CAT-mimetic activities, possibly due to their different oxidation states [116]. Nevertheless, in acidic inflammatory microenvironments, frequently used Mn-contained nanozymes tend to release large amounts of Mn^2+^, which can cause Fenton-like reactions and damage cells or tissues. Xiong et al. designed a new type of manganese-loaded mesoporous silica nanozyme (MnMSN) with KMnO_4_ oxidation surfactant templates [129]. Due to the presence of Mn^2+^ and Mn^4+^, MnMSNs can catalytically scavenge H_2_O_2_, ·OH, and ·O_2_^–^. MnMSNs suppress NF-κB activation by scavenging excessive ROS from inflammatory cells (M1 macrophages) and decrease proinflammatory mediator (TNF-α and IL-1β) production. In addition, Yao et al. prepared Mn_3_O_4_ nanoparticles (NPs) via a hydrothermal method, and these NPs prominently scavenge H_2_O_2_, ·OH, and ·O_2_^–^ better than CeO_2_ NPs and efficiently inhibit ROS-mediated ear inflammation in live mice [116]. Similarly, Ai et al. prepared multishelled manganese dioxide-encapsulated selenium–melanin (Se@Me@MnO_2_) nanocomposites through simple radical polymerization and the in situ oxidation‒reduction reaction method [130]. The Se@Me@MnO_2_ nanocomposites also function as powerful mimetics of multiple enzymes, including CAT, SOD, and GPx, to scavenge ROS in vitro, reducing ear inflammation in Kunming mice.

Hu et al. developed an adhesive hydrogel by combining polyvinyl alcohol (PVA), 3,4-dihydroxy-d-phenylalanine (DOPA) and MnO nanoparticles (NPs), named the PDMO hydrogel [131]. In addition to scavenging ROS and alleviating hypoxia in inflammatory microenvironments, the hydrogel exerts excellent antibacterial and antibiofilm effects. The PDMO hydrogel demonstrates significant therapeutic efficacy in alleviating gingivitis and periodontitis in Sprague‒Dawley rats, and its effects are even comparable to or better than those of PERIO, which is commercially available. The biosafety of the PDMO hydrogel was comprehensively investigated, and it was proven that the hydrogel has good biocompatibility, indicating that the PDMO hydrogel has great potential for future clinical translation.

##### Copper (Cu)-contained nanozymes

Copper, a necessary trace element in humans, plays a crucial role in the functions of many enzymes, including tyrosinase and SOD1. As a result, it is rational that Cu-contained nanozymes can scavenge ROS. Liu, T. et al. developed ultrasmall Cu_5.4_O NPs (Cu_5.4_O USNPs) with ROS scavenging ability due to their inherent ability to mimic multiple enzymes, such as CAT, GPx, and SOD [132]. Cu_5.4_O USNPs can be applied to treat diverse ROS-induced inflammatory conditions, including acute kidney injury (AKI), ALI, and wound healing both in vitro and in vivo. Peng et al. prepared Cu_5.4_O@Hep-PEG hydrogels with improved performance by adding star-shaped polyethylene glycol and heparin into Cu_5.4_O USNPs [133]. Cu_5.4_O@Hep-PEG hydrogels exhibit properties similar to CAT, GPx, and SOD scavenging ROS, preventing further inflammation-activation signaling.

In addition, Pt-contained nanozymes [134–136], Prussian blue-contained nanozymes [137–139] and carbon dot nanozymes [140, 141] have all been experimentally demonstrated to mimic enzymes that scavenge ROS. They are also promising biomaterials for the treatment of inflammatory diseases by targeting ROS.

#### Artificial selenoenzymes

GPx is a selenoenzyme that protects cells from ROS-induced damage. Artificial selenoenzymes with GPx activity have been prepared and applied to catalyze the reduction of hydroperoxides and reestablish physiological ROS homeostasis during treatment. Based on their structure, these artificial selenoenzymes are divided into two categories: selenoenzymes with direct Se-N–Se-O bonds and selenoenzymes with intramolecular noncovalent Se···N–Se···O bonds [142].

Diphenyl diselenide [(PhSe)_2_], an organoselenium compound with GPx-mimetic activity, exhibits anti-ROS and anti-inflammatory properties and has been broadly investigated in recent decades [143, 144]. (PhSe)_2_ has been proven to suppress histological inflammatory markers and ROS levels, thus inhibiting the NLRP3 inflammasome pathway and the IκB/NF-κB pathway [143, 144]. Several experiments have suggested that (PhSe)_2_ is a potential treatment for chronic infection in the brain caused by *Toxoplasma gondii* [143], ischemia/reperfusion insult [145] and amyotrophic lateral sclerosis (ALS) [144].

Ebselen [2-phenyl-1,2-benzisoselenazol-3(2H)-one] is another artificial selenoenzyme with GPx-mimetic activity. Xu et al. suggested that ebselen may inhibit the ROS generation induced by *H. pylori* LPS and alter the generation of IL‑8 by decreasing the phosphorylation of p38 MAPK [146]. Consequently, ebselen has shown strong potential for treating *H. pylori* infection. Chen, D. et al. demonstrated that ebselen can alleviate the influenza A virus-induced production of ROS and subsequent inflammatory responses [147]. Tewari et al. suggested that ebselen also decreases the excessive ROS production of TNFα-treated glioma cells [148]. This downregulation can reduce the production of the proinflammatory factors IL-6, IL-8, monocyte chemoattractant protein 1 (MCP-1), and COX-2 to prevent the establishment of a deleterious proinflammatory tumor microenvironment.

Guided by the successful design of ebselen, many ebselen derivatives, cyclic selenate esters, spirodioxyselenuranes, and various organotellurium compounds have been broadly reported [149]. However, the application of these biomaterials in clinical treatment still needs further research and improvement because of the significant toxic effects that are caused by these biomaterials [150].

Although the catalytic efficiency of natural antioxidant enzymes is better than that of nanozymes, their poor stability, high cost, and catalytic activity that is sensitive to environmental conditions have always limited their clinical application. As a new artificial enzyme with great potential, nanozymes combine the function of natural enzymes with the characteristics of nanomaterials and have the advantages of better cost, stability, and feasibility. These materials are currently considered to have potential for clinical therapeutic application in the future, but the problem of poor substrate selectivity still needs to be solved.

### Direct ROS scavenging biomaterials

#### Polyphenols

Polyphenols, as the name suggests, have multiple phenol rings. Polyphenols have been broadly utilized as exogenous ROS scavenging biomaterials and studied for possible use in the treatment or prevention of several ROS-related inflammatory diseases. Previous studies have shown that polyphenols can sacrificially react with free radicals and nonradicals. However, an increasing number of studies have revealed that the ROS scavenging effects of polyphenols occur due to their ability to react with free radicals, chelate metal catalysts, activate antioxidant enzymes, and inhibit oxidases.

##### Curcumin

Curcumin[1,7-bis(4-hydroxy-3-methoxyphenyl)-1,6-heptadiene-3,5-dione] can scavenge free radicals, including RNS and ROS. The free radical scavenging process relies on donation of a H-atom [151], which can be initiated by the phenolic OH group or the CH_2_ group of the b-diketone moiety. Free radicals perform electron transfer or extract H-atoms from these sites under normal conditions. Additionally, some specific biochemistry methods can inhibit the reaction, for instance, the addition of a phenolic OH group [152].

Researchers have developed various biomaterials based on curcumin to take advantage of its antioxidant and anti-inflammatory properties. First, curcumin has been employed to treat inflammatory diseases in the central nervous system. For instance, curcumin can improve neurological function in experimental rats with early brain injury after subarachnoid hemorrhage [153]. To overcome the limitations of curcumin’s low aqueous solubility and oral bioavailability, other studies have recently considered curcumin-loaded PLGA nanoparticles (Nps-Cur) and curcumin-loaded liposomes. Both of these approaches proved to be promising strategies for promoting neuroprotection against oxidative damage in AD [154, 155]. In addition, Qian, F. et al. applied hydrogel-embedded curcumin (TM/PC), which significantly reduces ROS levels and improves nerve regeneration and recovery after TBI [156]. Second, curcumin has excellent potential in wound healing. Chen et al. developed a curcumin-loaded sandwich-like nanofibrous membrane (CSNM) that exhibits strong antioxidant activity in DPPH radical scavenging tests [157]. Hu et al. synthesized a new type of hydrogel based on curcumin (OHA-CMC/CNP/EGF) [158]. They found that this hydrogel can decrease inflammation by releasing curcumin in the early stage of wound healing in a model of diabetic full-thickness skin wounds. Zhang, X. et al. described a glycosaminoglycan-based hydrogel delivery system that encapsulates curcumin [159]. This system exhibits strong ROS scavenging and anti-inflammatory properties and thereby accelerates chronic wound healing by regulating the wound microenvironment. Third, increasing attention has been given to the protective role of curcumin in ischemia‒reperfusion injury in animal models and various organs [160]. In addition, curcumin has shown promise in the treatment of many other ROS-induced inflammatory diseases, including AKI [161], ALI [162], and ankle inflammation [151].

##### Polydopamine (PDA)

PDA is an artificially synthesized form of melanin that has robust antioxidant activity. The ROS scavenging mechanism of PDA is not well understood due to its complicated structure, but the mechanism might be related to the molecular structure’s redox activity, inner radical lifetime, and superfast energy transfer via ion binding [163]. The catechol groups on PDA can quench free radicals and decrease the levels of certain compounds by providing H-atoms to the phenolic hydroxyl group. Then, a stable quinone structure is formed via the interaction between the resulting phenoxyl radicals and the second quenching free radicals. PDA has a mid-physiological range of redox potential, and its redox function can be repeated. The redox activity of PDA makes it possible to accept electrons from ascorbic acid and to donate electrons to ROS. In addition to the catechol groups, semiquinone radicals and oxidized o-quinones can also be involved in redox reactions [164]. Other studies have suggested that the key to the antioxidant activity of PDA is the rapid reduction of the o-quinone moiety in PDA to catechol by H_2_O_2_ radicals (HOO·) through a mechanism involving the transfer of H-atoms [165]. Moreover, the scavenging of ROS by PDA may also be associated with its strong ability to chelate metal ions and its SOD-like activity, which are related to the stable free radicals that “reside” within melanin [165–167].

As an excellent spectral ROS scavenger, PDA has been widely used to treat ROS-related inflammatory diseases. Bao et al. used PDA NPs in periodontal disease and found them to be effective in eliminating ROS and decreasing periodontal inflammation as potent antioxidants without causing side effects [168]. Fu et al. utilized reduced PDA loaded in hydrogel dressings to enhance its antioxidant activity to accelerate wound healing [169]. Battaglini et al. applied lipid-coated PDA NPs (L-PDNPs) to treat neurological diseases and observed both antioxidant and photothermal effects [170]. The results demonstrated that L-PDNPs effectively blocks ROS-induced mitochondrial dysfunction and accelerates the recovery of neurites. Zheng et al. developed PDA-wrapped manganese ferrite NPs (PDA@MF NPs) for the treatment of AKI [166]. The MF NPs constantly generate O_2_ in an H_2_O_2_-based hypoxic environment, which can polarize macrophages toward the M2 phenotype. PDA NPs function as ROS scavengers during O_2_ generation, contributing to reduced renal inflammation. Yan et al. constructed LS@PDA NPs with high anti-inflammatory and antioxidant activities, which can efficiently scavenge ROS and decrease the levels of proinflammatory cytokines, thus alleviating colonic inflammation in the treatment of IBD [171].

##### Tannic acid (TA)

TA is another type of plant-derived polyphenol with anti-inflammatory, anti-ROS, and antimicrobial properties [172]. Its large catechol groups make TA an innate free radical scavenger. The pyrogallol structure can be oxidized to form a quinone structure, which supplies hydrogen for TA and therefore resists oxidation. In addition to being rich in catechol and pyrogallol groups, TA can chelate diverse metal ions through coordination bonds and rapidly form a steady five-element ring complex with these ions.

Increasing numbers of studies have shown that TA and TA-based biomaterials have great potential in the treatment of different inflammatory and related diseases that are associated with ROS overproduction. Ni et al. obtained a TA-conjugated NP hydrogel (PPBA-TA-PVA) that can effectively function as an ROS scavenging agent and reduce inflammation through decreased proinflammatory mediator (IL-6and IL-1β) production and increased gene expression (TGF-β1, COL-1, COL-3) [173]. Li, Y. et al. constructed TA-chelated Fe-decorated molybdenum disulfide nanosheets (MoS@TA/Fe NSs) that were fixed to multifunctional hydrogels [174]. Benefitting from the TA/Fe complex, the hydrogels decompose HO into O in a neutral environment to cope with hypoxia by supplying appropriate oxygen. Additionally, this agent showed a strong ability to scavenge ROS and RNS and decrease the production of inflammatory mediators to maintain antioxidant system homeostasis and prevent inflammation. Shi et al. constructed a multifunctional HA–PBA–TA dynamic hydrogel, which was proven to have good ROS scavenging properties [175]. By introducing TA into the quaternized chitosan (QCS) matrix, Pan et al. generated a new type of hydrogel featuring strong ROS scavenging and antibacterial properties [176]. The novel biomaterials described above provide promising strategies for promoting wound healing in future clinical practice. Recently, TA-based biological materials have been increasingly studied in the context of IBD [177, 178], liver injury prevention [179], ischemic stroke [180], cognitive impairment [181], anti-UV skin protection [182], bone renovation and regeneration [183–185] and OA [186].

#### Hydrogen-based materials

Hydrogen (H_2_) has attracted extensive attention as an effective ROS scavenging biomaterial. In fact, H_2_ can selectively reduce highly cytotoxic ROS concentrations in diseased cells while preserving the physiological functions of ROS in normal cells. Furthermore, H_2_ can quickly diffuse into target cells and tissues to perform its therapeutic functions since it is smaller than other antioxidants. It can also pass through the blood‒brain barrier (BBB), while most ROS scavenging biomaterials cannot. Because of its unique property of scavenging ROS without causing side effects, H_2_ is a promising strategy for treating ROS-induced inflammation [187, 188].

Currently, many mechanisms of the antioxidant effect of H_2_ have been proposed, the most important of which is the direct mechanism. That is, H_2_ chemically reacts with ROS, including ·OH. The direct mechanism of ·OH scavenging is achieved by the chemical reaction of H_2_ + ·OH → H_2_O + H· followed by H· + O_2_^−^  → HO_2_^−^ [189]. However, this interpretation remains partially flawed from the atomic perspective [187]. The direct chemical reaction of H_2_ with ROS has only been demonstrated in acellular experiments [189]. Mild H_2_ levels can scavenge a variety of ROS in vivo, suggesting that either H_2_ is cleaved into H-atoms by intracorporal enzymes with strong activity or the chemical structure of H_2_ is altered by external effects that break the bond between H-atoms. Kim et al. demonstrated that protoheme, including the transition metal Fe, might mediate this process, effectively reducing the activation barrier of hydrogen molecules [187]. In addition, H_2_ can also significantly scavenge ROS by activating the Nrf2 pathway, promoting the expression of antioxidants and downregulating the level of NOX-2 [187, 189, 190].

During inflammation, H_2_ decreases the infiltration of neutrophils and M1 macrophages and reduces the release of proinflammatory mediators, including IL-1β, IL-6, IL-8, IL-10, TNF-α and interferon-γ [190]. Specifically, in an airway disease model, H_2_ recovered H_2_O_2_-induced and LPS-induced ROS generation and suppressed MAPK activation in A549 and NCI-H292 cells in vitro [191]. Similarly, in a periodontitis model, H_2_ treatment decreased IL-1α and IL-6, important cytokines related to inflammation in periodontal tissues [192]. In addition, H_2_ has shown good therapeutic potential in ROS-induced inflammatory diseases such as ALI [193], COVID-19 [194], psoriasis-associated skin lesions, and arthritis [195].

To maximize the anti-inflammatory effects of H_2_, Wan et al. developed a multicomponent nanoreactor (NR) that comprises chlorophyll a, l-ascorbic acid, and gold nanoparticles that can generate H_2_ in situ upon photon absorption, such as photosynthesis, in plants [196]. Their results confirmed that the novel system can reduce excessive levels of ROS and proinflammatory mediators and successfully ameliorate foot inflammation in mice. Recently, the team has prepared a similar system for photocatalytic H_2_ production, adding the capability of simultaneous imaging to the initial anti-inflammatory treatment [197].

#### Sulfur-based oxidation-responsive biomaterials

Sulfur is a vital element with excellent biocompatibility in biological systems [198]. Sulfur compounds are currently considered important tools in the treatment and prevention of inflammatory diseases because of their ROS scavenging properties.

Sulfur mainly participates in ROS scavenging through the Trx system, which is characterized by the conserved amino acid sequence Trp-Cys-Gly-Pro-Cys. In this sequence, Cys32 and Cys35 are redox-active and are apt to be oxidized by ROS. The oxidized Trx, in which disulfide bonds are formed between two thiol moieties, can receive electrons from NADPH, thereby being reduced to reactive Trx with the assistance of TrxR. According to this reaction, Trx undergoes a cyclic redox reaction to ensure the persistence of its ROS scavenging abilities [199, 200].

##### Thioether/sulfoxide-contained biomaterials

Thioethers are a class of sulfur-contained compounds featuring an R–S–R′ moiety, in which R and R′ represent alkyl or aryl groups [201]. These compounds are readily oxidized into sulfoxide or sulfone by relatively high concentrations of ROS [202]. Therefore, polythioether/polysulfide can directly function according to an anti-inflammatory principle. Immediate clearance of ROS has been shown to inhibit inflammatory pathways in mice hours after ischemic stroke and significantly reduce systemic administration (as well as brain damage due to accumulation of drugs in the brain due to BBB damage) [203]. In addition, a similar anti-inflammatory effect was observed in mice with TBI after the injection of thioether cross-linked polysorbate nanoparticles [204]. Similarly, poly(propyl thipropane) particles reduce inflammatory tissue damage in diabetic mice with ischemic limb injury and mechanical cartilage injury after local injection [205]. Absorbable thioether-grafted HA nanofibrous hydrogels reduce wound inflammation and facilitate wound healing in diabetic models compared to unmodified HA [206].

Thioethers are hydrophobic structures, while sulfoxide and sulfone have more vital polarity; thus, thioethers undergo a change from hydrophobicity to hydrophilicity when oxidized, which leads to an obvious increase in their solubility in the aqueous environment [202]. Therefore, polythioether/polysulfide can be used as a carrier material to achieve good ROS responsiveness, and the addition of anti-inflammatory drugs can exert a synergistic effect between ROS clearance and pharmacological drug activity.

When thioethers are oxidized to sulfoxide, in which the sulfur atom has a lone electron pair, some sulfoxides, including dimethyl sulfoxide (DMSO), can continue to be oxidized to sulfone by ROS [198, 201]. DMSO can be used as a drug, for example, in treating interstitial cystitis [207]. In fact, DMSO shows significant anti-inflammatory properties and inhibits lymphocyte activation as well as M1 or M2 macrophage polarization [208]. In addition, it can resolve rheumatoid arthritis in mice at concentrations of less than 10 vol% (70 vol% local administration). These properties are believed to be due to DMSO’s ROS scavenging ability, which may be secondary to the redox reaction of thioethers [209]. Another small molecule, sulfoxalicin (s-allyl-l-cysteine sulfoxide, found in garlic), is also shown to have antioxidant and anti-inflammatory properties.

##### Thioacetals/thioketals-contained biomaterials

Thioacetals/thioketals are also ROS scavenging materials containing sulfur, and their activity is derived from the condensation reaction of mercaptans with aldehydes/ketones. They can be oxidized and cleaved by ROS while remaining relatively stable in acidic and alkaline environments [202].

Dual pH- and ROS-responsive nanomaterials have been constructed from the conanocrystalline precipitation of hydrophobically modified chitosan, polythioketone, and curcumin. The nanomaterials successfully inhibit the proinflammatory pathway by scavenging ROS (thioketone and curcumin) in ankle inflammation [151]. Due to the properties of polythioneone, it can also be applied in ROS-degradable implants. For instance, polythioneone diol reacts with triisocyanate to form polythioneone polyurethane scaffolds, which are degraded by ROS. Currently, these agents have been used in ischemic wound healing, and composite materials containing these materials and hydroxyapatite have been used to repair bone defects.

##### Hydrogen sulfide

Hydrogen sulfide (H_2_S) is a toxic and corrosive gas with the characteristic odor of rotting eggs. H_2_S has a structure similar to a water molecule and is readily oxidized into a variety of forms, including elemental sulfur, sulfate (SO_4_^2–^), thiosulfate (S_2_O_3_^–^), and sulfur dioxide (SO_2_) [210].

H_2_S can remove ROS and RNS more easily and quickly than traditional scavengers [211]. Although the activity of H_2_S as a physiological antioxidant has been questioned, exogenous H_2_S has shown excellent protective abilities when cells are exposed to ROS. In addition, H_2_S can freely diffuse across cell membranes due to its low molecular weight [212].

H_2_S, acting as an antioxidant, shows therapeutic potential in several inflammatory diseases by eliminating the ROS that is generated during pathological processes. H_2_S may play a significant role in treating cardiovascular diseases, neurodegenerative diseases, and other inflammatory diseases. H_2_S can potentially protect cardiac tissues against cardiovascular diseases, possibly by reducing oxidative stress and maintaining cell apoptosis of the endothelial cells. Endothelial cells are found inside blood and lymphatic vessels, and their disorder is thought to be associated with a range of cardiovascular diseases, such as atherosclerosis and hypertension [210]; inflammatory responses are the main pathological processes of these diseases. H_2_S was first identified as a neuromodulator before its other functional roles were identified. Evidence of congenital inflammatory responses in AD was suggested 20 years ago. Subsequent studies have also revealed a role of inflammation in Parkinson’s disease (PD), ALS, multiple sclerosis (MS), and an increasing number of other central nervous system diseases [213]. Treatment with H_2_S has been proven to decrease the level of ROS and cognitive impairment in APP/PS1 mouse models of AD, thus effectively treating AD.

#### Bilirubin-derived biomaterials

Bilirubin, an endogenous antioxidant, is mainly produced by the breakdown of heme. As a kind of tetrapyrrole, the antioxidative effect of bilirubin is closely related to its structure. Specifically, a fully exposed hydrogen atom attached to the C-10 bridge can combine with the outermost lone pair electron of oxygen radicals and thus directly scavenge ROS [214]. In addition, the bilirubin–biliverdin cycle further promotes the antioxidant activity of bilirubin [215]. Moreover, bilirubin triggers the intranuclear translocation of Nrf2 and enhances the expression of HO-1 and antioxidants, regenerating bilirubin and activating various antioxidant enzymes, and thus reducing ROS levels through intranuclear regulation [216, 217].

By modifying bilirubin or loading bilirubin in nanocarriers, multiple bilirubin-derived nanoparticles (BRNPs) have been constructed to achieve high water solubility, favorable stability and advanced efficacy. To date, BRNPs have been proven to have promising prospects for application in various disease models. Kim, Dong Eon et al. established a mouse model of asthma and proved that BRNPs can inhibit Th2-mediated lung inflammation partially by scavenging ROS in CD4 + T cells [218]. Yao, Q. et al. constructed bilirubin-encapsulated silk fibrin nanoparticles (BRSNPs) for the treatment of acute pancreatitis and found that both bilirubin and BRSNP can markedly ameliorate ROS concentrations, and BRSNP performed better in scavenging ROS [219]. In osteoarthritis, Xue, Song et al. indicated that BRNPs prevent cartilage degeneration by scavenging ROS, promoting autophagy and inhibiting the NF-κB pathway in an anterior cruciate ligament transection rat model [220]. Studies that focused on ulcerative colitis have adopted various BRNPs, including hyaluronic acid–bilirubin nanoparticles (HABNs) [221], PEGylated bilirubin micelles [222], and bilirubin self-assembled nanomedicine (BSNM) [223], all of which have demonstrated good ROS responsiveness and scavenging activity. Studies have also shown that BRNPs can be powerful ROS scavengers to manage cardiac and hepatic ischemia–reperfusion injuries [224, 225]. Choi, Chong Won et al. have shown that BRNPs principally accumulate in the inflamed site where they reduce ROS levels and thus contribute to therapeutic effects in an LL-37-induced rosacea-like mouse model. Several studies have also focused on psoriasis, another chronic inflammatory skin disease, and found that BRNPs induce a concentration-dependent reduction in both intracellular and extracellular ROS levels [226, 227]. Pulmonary fibrosis has attracted increasing attention in recent years as a sequela of severe coronavirus pneumonia. To address this conditions, Keum, Hyeongseop et al. applied PEGylated bilirubin micelles to a mouse model, which were proven to preferentially assemble in inflamed lesions, reducing oxidative stress and markedly attenuating symptoms [228].

In summary, the ROS scavenging biomaterials that exert direct effects that we discussed above exhibit outstanding antioxidant, anti-inflammatory, and ROS scavenging capacities. Their favorable biocompatibilities also contribute to their practical applications; of these materials, endogenous bilirubin-based biomaterials might perform better than other exogenous agents. For their distribution in vivo, biomaterials, including H_2_, BRNPs, etc., are more likely to aggregate in target tissues in different ways, and studies are focusing on modifying biomaterials to facilitate their more precise transport to inflamed lesions. Further studies are needed to concentrate on how to generate updated biomaterials that possess maximized anti-inflammatory ability while also having excellent stability, solubility, biocompatibility, site specificity, and low production cost.

### ROS generation-blocking biomaterials

#### NOX-inhibiting biomaterials

Natural biomaterials have been widely investigated by many scholars in recent years, and the understanding of natural biomaterials is gradually becoming clearer. Some of these natural biomaterials, such as apocynin, can inhibit ROS production by inhibiting NOX activity or NOX-mediated ROS production, thus achieving anti-inflammatory effects. For example, apocynin inhibits the production of ROS by inhibiting NOX-2. Thus, the phosphorylation activity of p38 MAPK is inhibited. This compound blocks the transport of p47phox to the membrane and reduces NOX-2-dependent ROS production, which plays a protective role in some inflammasome experimental models [229]. As it is nontoxic and can reduce markers of oxidative stress, the compound is widely used in animal models of inflammasome diseases, including collagen-induced arthritis, and it has significant preventive properties. Similarly, apocynin plays a significant role in preventing inflammation in models of ulcerative colitis and asthma [230]. In addition, Qin et al. demonstrated that cotreatment with apocynin and NADPH significantly reduces infarct volume, improves poststroke survival, and can restore neurological function in a mouse model of stroke [231]. Other natural biomaterials are also associated with inhibiting NOX, such as celastrol and Honokiol. However, the relationship between these natural biomaterials and ROS inhibition or anti-inflammation is still unclear. Therefore, further studies on their specific mechanism are needed.

In addition to natural biomaterials, artificial biomaterials can also block the production of ROS by inhibiting NOX. Diphenyliodonium (DPI) is a specific and effective inhibitor of NOX that can reduce the activity of NOX and inhibit the production of ROS. The intracellular ROS levels, the number of inflammatory cells, and the levels of cytokines in DPI-treated rats with ALI were significantly reduced, which suggests that DPI can be used as a potential anti-inflammatory agent in ALI [232].

#### XO-inhibiting biomaterials

Similarly, natural biomaterials account for an important proportion of XO-inhibiting biomaterials. *Stevia rebaudiana* is a stevia plant of the *Stevia* genus in the Compositae family, and its extracts can exert antihyperglycemic, antihypertensive, anti-inflammatory, and antitumor effects and prevent acute and chronic liver injury, diuresis, and immune regulation. Mehmood et al. prepared stevia residue extract (STVRE) that was rich in flavonoids and chlorogenic acid [233]. In vitro results showed that the IC50 value of STVRE in inhibiting XO is 8.78 ± 0.89 μg·mL^−1^. In vivo STVRE has a beneficial effect on mice with hyperuricemia induced by fructose combined with potassium oxyazinate, inhibiting XO activity and improving oxidative stress and the inflammatory response. Mulberry leaves are the dried leaves of *Morus alba*, a plant belonging to the Moraceae family. The main active ingredients in mulberry leaves are flavonoids, alkaloids, phytosterols, gamma-aminobutyric acid, and polysaccharides, which lower blood pressure, lower blood glucose and lipids, and exert anti-inflammatory and antitumor effects. Wan et al. found that ethanol extracted from mulberry leaves could competitively inhibit XO activity in a dose-dependent manner [234]. This effect still needs to be verified by in vivo experiments, and the specific active ingredients need to be further studied.

In addition to natural biomaterials, it is believed that endogenous corticosterone can inhibit the expression of XO and thus inhibit inflammation. Wu et al. found that a higher corticosterone concentration (higher than 700 μg/L) downregulated NLRP3 expression within 2 h of inflammation, alleviated the inflammatory response in mouse macrophages, and inhibited the expression level of XO. The results showed that a higher concentration of corticosterone (higher than 700 μg/L) inhibits NLRP3 expression in mouse macrophages [235].

#### LOX-inhibiting biomaterials

At present, research on LOX-inhibiting biomaterials is limited to inhibitors of different LOX subtypes. Cerqua et al. found that indole compounds can act as dual inhibitors of 5-LOX/sEH, showing significant anti-inflammatory effects in mice with glycan-induced peritonitis and experimental asthma in vivo [236]. The results provide a basis for using 5-LOX/sEH dual inhibitors as anti-inflammatory agents. Nagesh Khadri et al. found that (benzoylphenoxy)-N-[237] can act as an anti-inflammatory COX/5-LOX inhibitor [237].

A novel dual pH/REDOX reactive polymer nanoliposome system (NL) loaded with copper ligand bioactive complexes has been designed as a controlled delivery system for the management of inflammation [238]. The NL was synthesized after preparation of the copper–glyglycine–prednisolone succinate ([(Cu(glygly)(PS)]) complex and the dual pH/redox responsive biopolymer. The prednisolone succinate [Cu(glygly)(PS)] complex demonstrates significant performance in scavenging free radicals and inhibiting LOX-5. The results indicated that this novel cupric ligand bioactive drug delivery system has a controllable drug release mechanism and can be used as a potential drug delivery system for treating inflammation.

#### ETC-regulating biomaterials

In addition to eliminating ROS by using Ce3 + and Ce4 + as SOD and CAT mimics through recyclable changes between CE3 + and CE4 + , CeO_2_ NPs can also inhibit ROS production through an effect on the METC. Li et al. found that 1–100 μg/ml CeO_2_ NPs effectively reduces superoxide flux in the METC in a concentration-dependent manner [239]. Succinic acid-driven mitochondria isolated from macrophages also showed the inhibitory effects of the nanogranules. The results indicated that CeO_2_ NPs can effectively reduce the superoxide flux from METC in human macrophages, which may have important implications for protecting against inflammatory disease processes. Stahr et al. discovered that Hst nanocrystals exert strong antioxidative effects according to an DPPH assay [240]. Hst, a flavonoid, is the aglycon of hesperidin. HstP is extracted from hesperidin by hydrolysis and can prevent mitochondrial dysfunction. HstP can reduce cell viability, prevent ROS formation, increase CAT activity, and prevent the reduction in mitochondrial membrane potential induced by H_2_O_2_. Brain aging and age-related neurodegenerative disorders are closely related to mitochondrial dysfunction. It is believed that mitochondrial dysfunction is a marker of these neurological disorders. For example, in AD, mitochondrial dysfunction is characterized by an impaired ETC, reduced levels of adenosine triphosphate (ATP), and elevated generation of ROS. Babylon et al. found that Hst nanocrystals are more beneficial in preventing mitochondrial dysfunction in a cellular model of early AD than pure Hst [241].

In conclusion, biomaterials that prevent ROS production are a class of biomaterials that scavenge ROS effectively by blocking ROS generation at the source. However, this new type of biomaterial lacks tissue specificity and is unable to exert therapeutic effects due to the low bioavailability in specific organs. In addition, how to ensure that inhibiting ROS production does not affect the physiological roles of ROS still needs further research.

## Challenges and perspectives

ROS scavenging biomaterials undoubtedly assist in treating inflammatory diseases by inhibiting ROS-mediated inflammation. However, many potential challenges and critical issues must be addressed in order to apply ROS scavenging materials that target oxidative stress to treat inflammatory diseases. For instance, there is still a lack of global and systematic understanding of the roles of ROS in inflammation. Different doses of ROS, different sources of ROS, or different sites of action result in different effects, which indicates that our understanding of ROS is still not complete [242]. The reason may be that current studies concentrate on a single pathway or molecule while ignoring the causal relationship between ROS and inflammation and the complex regulatory mechanisms involved. Further research needs to focus on the network of ROS to understand the multiple roles of ROS in inflammation from a more complete perspective; such studies will contribute to the design of anti-inflammatory biomaterials and the understanding of their working mechanisms.

In addition, targeting biomaterials is a focus of current research. Currently, some material systems target lesion sites by utilizing the electrical charges of different tissues, changing the injection/administration methods, and setting initiators and other spatiotemporal controls, which should be advocated [243, 244]. However, for most ROS scavenging biomaterials, targeting and establishing evaluation systems remains an urgent problem. On the one hand, we need to determine how to accurately deliver biomaterials to organs or tissues with dysregulated inflammatory responses. On the other hand, ROS from different sources may play different roles in the inflammatory response [171, 196, 197, 245], requiring biomaterials to precisely target ROS that negatively affect the body. The targeting of anti-inflammatory therapies to reduce side effects and improve treatment efficiency is likely to be a focus of future research.

It should also be noted that low levels of ROS are necessary for biological activities, and they are important signaling molecules that regulate several physiological functions, including inflammatory responses and metabolism [244]. Therefore, ROS scavenging biomaterials should aim to store the levels of ROS to physiological equilibrium as much as possible rather than completely scavenge ROS, which would cause a certain degree of damage to the body. In the biosafety field, it is necessary to monitor the normal biochemical processes mediated by ROS after the application of biological materials.

## Conclusion

We summarize recent advances in treating inflammatory diseases with ROS scavenging biomaterials. The balance between ROS production and elimination under physiological conditions, the relationship between ROS and inflammatory signaling pathways, and the application of biomaterials with different ROS scavenging mechanisms in inflammatory diseases are comprehensively reviewed to solve some problems that may be faced in this emerging field. When the body is exposed to various adverse stimuli and the physiological homeostasis of ROS is disrupted, excessive ROS act as signaling molecules and toxic substances to activate inflammatory responses. Based on this, we reviewed ROS scavenging biomaterials that function by blocking the production of ROS, directly reacting with ROS, and accelerating the removal of ROS through catalysis. In this review, the mechanisms underlying ROS scavenging and the applications of these biomaterials in inflammatory diseases are introduced in detail.

In conclusion, the current development of ROS scavenging biomaterials for the treatment of inflammatory diseases has been rapid, offering countless possibilities for biomedicine as a promising therapeutic modality. Although some problems need to be carefully solved, the great possibilities of these biomaterials deserve further exploration. We hope this review will provide adequate information for the further development of this field, which would facilitate the more rapid development of anti-inflammatory biomaterials in the future.

## Data Availability

Not applicable.

## Abbreviations

ROS: Reactive oxygen species
COVID-19: Coronavirus disease 2019
·OH: Hydroxyl radical
O_2_^−^: Superoxide anion
^1^O_2_: Singlet oxygen
H_2_O_2_: Hydrogen peroxide
IBD: Inflammatory bowel diseases
COPD: Chronic obstructive pulmonary disease
OA: Osteoarthritis
METC: Mitochondrial electron transport chain
MIM: Mitochondrial inner membrane
NOX: Nicotinamide adenine dinucleotide phosphate oxidases
XOR: Xanthine oxidoreductase
LOX: Lipoxygenase
DUOX: Dual oxidases
NADPH: Nicotinamide adenine dinucleotide phosphate
XDH: Xanthine dehydrogenase
XO: Xanthine oxidase
AA: Arachidonic acid
PUFAs: Polyunsaturated fatty acids
IR: Ionizing radiation
LET: Low linear energy transfer
SOD: Superoxide dismutase
CAT: Catalase
GPx: Glutathione peroxidase
SOD1: Cytosolic CuZn-SOD
SOD2: Mitochondrial Mn-SOD
SOD3: Extracellular SOD
GSH: Glutathione
mtROS: Mitochondrial ROS
NF-κB: Nuclear transcription factor-kappa B
NLRP3: Nod-like receptor family pyrin domain-containing 3
PRRs: Pattern-recognition receptors
PYD: Pyrin domain
NACHT: Central nucleotide-binding or oligomerization domain
LRRs: Leucine-rich repeats
pro-IL-1β: Pro-interleukin-1β
DN: Diabetic nephropathy
TRAPS: Tumor necrosis factor receptor-associated periodic syndrome
AD: Alzheimer’s disease
TRX: Thioredoxin
TXNIP: Thioredoxin-interacting protein
mtDNA: Mitochondrial DNA
IL-1β: Interleukin-1β
RHD: Rel homology domain
IKK: IκB kinase
COX-2: Cyclooxygenase-2
Th1: T-helper1
Tfh: T follicular
Jurkat JR: Jurkat cells
LPC: Lysophosphatidylcholine
PKD: Protein kinase D
NIK: NF-κB-inducing kinase
MAPK: Mitogen-activated protein kinase
ERKs: Extracellular signal-related kinases
p38: P38 kinase
JNKs: C-Jun N-terminal kinases
MAP3K: Mitogen-activated kinase kinase kinase
MAP2K: Mitogen-activated kinase
MAPKAPKs: MAPK-activated protein kinases
RSK: Ribosomal-S6-kinases
MSK: Mitogen- and stress-activated kinases
MNK: MAPK-interacting kinases
MK: MAPKAPKs
IFN-β: Interferon-β
TNF-α: Tumor necrosis factor-α
NAC: N-acetylcysteine
ROT: Rotenone
TRAF2: TNF receptor-associated factor 2
MLK3: Mixed lineage kinase 3
EGFR: EGF receptor
PDGFR: PDGF receptor
MKPs: MAPK phosphatases
JAK/STAT: Janus kinase/signal transducer and activator of transcription
PI3K: Phosphate kinase 3-kinase
RSV: Respiratory syncytial virus
Nrf2: Nuclear factor erythroid 2-related factor 2
CNC: Cap ‘n’ collar
Neh1-7: Nrf2-ECH homologous domains
sMaf: Small Maf
KEAP1: Kelch-like ECH-associated protein 1
ARE: AU-rich element
HO-1: Heme oxygenase-1
PKC: Protein kinase C
15d-PGJ2: 15-Deoxyd-d-prostaglandin J2
ALI: Acute lung injury
PKB/AKT: Protein kinase B
PIP2: Phosphatidylinositol 4,5-diphosphate
PIP3: Phosphatidylinositol 3,4,5-triphosphate
PH: Pleckstrin homology
PDK: Phosphoinositol-dependent protein kinase
PTEN: Phosphatase and tensin homology
Gel: Gelatin
Alg: Alginate
Gel–Alg: Gelatin–alginate biocompatible hydrogel
GelMA: Methacrylated gelatin
NPG: Nanoporous gold
γ-PGAS: γ-PGA with taurine
SOD-PGAS/PGA-H: SOD-loaded γ-PGAS/γ-PGA hydrogel
PP: Poly (N-isopropyl-acrylamide)/poly (gamma-glutamic acid)
HA: Hyaluronic acid
HT: Hyaluronic acid-tyramine
TBI: Traumatic brain injury
MA: Malonic acid
SHP: Peptides from seahorse hydrolysates
BG: Bioactive glasses
CeO_2_ NPs: Cerium oxide nanoparticles
Ce^3+^: Trivalent cerium atoms
Ce^4+^: Tetravalent cerium atoms
CCNs: Chitosan-coated CeO_2_ nanocubes
AMD: Age-related macular degeneration
SCI: Spinal cord injury
Mn-contained: Manganese-contained
MnMSN: Manganese-loaded mesoporous silica nanozyme
NPs: Nanoparticles
Se@Me@MnO_2_: Multishelled manganese dioxide-encapsulated selenium–melanin
Cu: Copper
Cu_5.4_O USNPs: Ultrasmall Cu_5.4_O NPs
AKI: Acute kidney injury
ALI: Acute liver injury
ALS: Amyotrophic lateral sclerosis
MCP-1: Monocyte chemoattractant protein 1
Nps-Cur: Curcumin-loaded PLGA nanoparticles
TM/PC: Hydrogel-embedded curcumin
CSNM: Curcumin-loaded sandwich-like nanofibrous membrane
PDA: Polydopamine
HOO·: H_2_O_2_ radical
L-PDNPs: Lipid-coated PDA NPs
PDA@MF NPs: Polydopamine-wrapped manganese ferrite nanoparticles
TA: Tannic acid
PPBA-TA-PVA: TA-conjugated nanoparticle hydrogel
MoS@TA/Fe NSs: TA-chelated Fe-decorated molybdenum disulfide nanosheets
QCS: Quaternized chitosan
H_2_: Hydrogen
BBB: Brain‒blood barrier
NR: Nanoreactor
DMSO: Dimethyl sulfoxide
H_2_S: Hydrogen sulfide
SO_4_^2–^: Sulfate
S_2_O_3_^–^: Thiosulfate
SO_2_: Sulfur dioxide
PD: Parkinson’s disease
MS: Multiple sclerosis
DPI: Diphenyliodonium
MOSCs: Metal–organic supercontainers
4-OI: 4-Octyl itaconate
B-NPs: Butyrate-supported chitosan/HA nanoparticles
SCFA: Short-chain fatty acid
STVRE: Stevia residue extract
NLs: Nanoliposome system
ATP: Adenosine triphosphate
MSC: Mesenchymal stem cells

## References

[1] Medzhitov R (2008). Origin and physiological roles of inflammation. Nature.

[2] Sun Y, Chen P, Zhai B, Zhang M, Xiang Y, Fang J (2020). The emerging role of ferroptosis in inflammation. Biomed Pharmacother.

[3] Raman B, Bluemke DA, Lüscher TF, Neubauer S (2022). Long COVID: post-acute sequelae of COVID-19 with a cardiovascular focus. Eur Heart J.

[4] D'Autreaux B, Toledano MB (2007). ROS as signalling molecules: mechanisms that generate specificity in ROS homeostasis. Nat Rev Mol Cell Biol.

[5] Yang B, Chen Y, Shi J (2019). Reactive oxygen species (ROS)-based nanomedicine. Chem Rev.

[6] Zhang J, Wang X, Vikash V, Ye Q, Wu D, Liu Y (2016). ROS and ROS-mediated cellular signaling. Oxid Med Cell Longev.

[7] Rendra E, Riabov V, Mossel DM, Sevastyanova T, Harmsen MC, Kzhyshkowska J (2019). Reactive oxygen species (ROS) in macrophage activation and function in diabetes. Immunobiology.

[8] Zhu H, Li YR (2012). Oxidative stress and redox signaling mechanisms of inflammatory bowel disease: updated experimental and clinical evidence. Exp Biol Med (Maywood).

[9] Lee IT, Yang CM (2012). Role of NADPH oxidase/ROS in pro-inflammatory mediators-induced airway and pulmonary diseases. Biochem Pharmacol.

[10] Ansari MY, Ahmad N, Haqqi TM (2020). Oxidative stress and inflammation in osteoarthritis pathogenesis: role of polyphenols. Biomed Pharmacother.

[11] Panchal NK, Prince SE (2023). Non-steroidal anti-inflammatory drugs (NSAIDs): a current insight into its molecular mechanism eliciting organ toxicities. Food Chem Toxicol.

[12] Wang L, Zhu B, Deng Y, Li T, Tian Q, Yuan Z (2021). Biocatalytic and antioxidant nanostructures for ROS scavenging and biotherapeutics. Adv Func Mater.

[13] Zou Z, Chang H, Li H, Wang S (2017). Induction of reactive oxygen species: an emerging approach for cancer therapy. Apoptosis.

[14] Zhao RZ, Jiang S, Zhang L, Yu ZB (2019). Mitochondrial electron transport chain, ROS generation and uncoupling (Review). Int J Mol Med.

[15] Alfadda AA, Sallam RM (2012). Reactive oxygen species in health and disease. J Biomed Biotechnol.

[16] Mailloux RJ, McBride SL, Harper ME (2013). Unearthing the secrets of mitochondrial ROS and glutathione in bioenergetics. Trends Biochem Sci.

[17] He L, He T, Farrar S, Ji L, Liu T, Ma X (2017). Antioxidants maintain cellular redox homeostasis by elimination of reactive oxygen species. Cell Physiol Biochem.

[18] Sedeek M, Nasrallah R, Touyz RM, Hebert RL (2013). NADPH oxidases, reactive oxygen species, and the kidney: friend and foe. J Am Soc Nephrol.

[19] Granger DN, Kvietys PR (2015). Reperfusion injury and reactive oxygen species: the evolution of a concept. Redox Biol.

[20] Minakami R, Sumimotoa H (2006). Phagocytosis-coupled activation of the superoxide-producing phagocyte oxidase, a member of the NADPH oxidase (nox) family. Int J Hematol.

[21] Bedard K, Krause KH (2007). The NOX family of ROS-generating NADPH oxidases: physiology and pathophysiology. Physiol Rev.

[22] Cho KJ, Seo JM, Kim JH (2011). Bioactive lipoxygenase metabolites stimulation of NADPH oxidases and reactive oxygen species. Mol Cells.

[23] Luchtefeld M, Drexler H, Schieffer B (2003). 5-Lipoxygenase is involved in the angiotensin II-induced NAD(P)H-oxidase activation. Biochem Biophys Res Commun.

[24] Kawamura K, Qi F, Kobayashi J (2018). Potential relationship between the biological effects of low-dose irradiation and mitochondrial ROS production. J Radiat Res.

[25] Wang Y, Branicky R, Noe A, Hekimi S (2018). Superoxide dismutases: dual roles in controlling ROS damage and regulating ROS signaling. J Cell Biol.

[26] Wendel A (1981). Glutathione peroxidase. Methods Enzymol.

[27] Kim DH, Meza CA, Clarke H, Kim JS, Hickner RC (2020). Vitamin D and Endothelial Function. Nutrients.

[28] Traber MG, Atkinson J (2007). Vitamin E, antioxidant and nothing more. Free Radic Biol Med.

[29] Li MS, Adesina SE, Ellis CL, Gooch JL, Hoover RS, Williams CR (2017). NADPH oxidase-2 mediates zinc deficiency-induced oxidative stress and kidney damage. Am J Physiol Cell Physiol.

[30] Salazar G, Huang J, Feresin RG, Zhao Y, Griendling KK (2017). Zinc regulates Nox1 expression through a NF-kappaB and mitochondrial ROS dependent mechanism to induce senescence of vascular smooth muscle cells. Free Radic Biol Med.

[31] Kelley N, Jeltema D, Duan Y, He Y (2019). The NLRP3 inflammasome: an overview of mechanisms of activation and regulation. Int J Mol Sci.

[32] Liu Q, Zhang D, Hu D, Zhou X, Zhou Y (2018). The role of mitochondria in NLRP3 inflammasome activation. Mol Immunol.

[33] Zhou R, Tardivel A, Thorens B, Choi I, Tschopp J (2010). Thioredoxin-interacting protein links oxidative stress to inflammasome activation. Nat Immunol.

[34] Place DE, Kanneganti TD (2018). Recent advances in inflammasome biology. Curr Opin Immunol.

[35] Groß CJ, Mishra R, Schneider KS, Médard G, Wettmarshausen J, Dittlein DC (2016). K(+) efflux-independent NLRP3 inflammasome activation by small molecules targeting mitochondria. Immunity.

[36] Han Y, Xu X, Tang C, Gao P, Chen X, Xiong X (2018). Reactive oxygen species promote tubular injury in diabetic nephropathy: the role of the mitochondrial ros-txnip-nlrp3 biological axis. Redox Biol.

[37] Bulua AC, Simon A, Maddipati R, Pelletier M, Park H, Kim KY (2011). Mitochondrial reactive oxygen species promote production of proinflammatory cytokines and are elevated in TNFR1-associated periodic syndrome (TRAPS). J Exp Med.

[38] Chen Y, Liu Y, Jiang K, Wen Z, Cao X, Wu S (2023). Linear ubiquitination of LKB1 activates AMPK pathway to inhibit NLRP3 inflammasome response and reduce chondrocyte pyroptosis in osteoarthritis. J Orthop Translat.

[39] Wang SY, Fu XX, Duan R, Wei B, Cao HM, Yan E (2023). The Alzheimer's disease-associated gene TREML2 modulates inflammation by regulating microglia polarization and NLRP3 inflammasome activation. Neural Regen Res.

[40] Dostert C, Pétrilli V, Van Bruggen R, Steele C, Mossman BT, Tschopp J (2008). Innate immune activation through Nalp3 inflammasome sensing of asbestos and silica. Science.

[41] An Z, Su J. Acinetobacter baumannii outer membrane protein 34 elicits NLRP3 inflammasome activation via mitochondria-derived reactive oxygen species in RAW264.7 macrophages. Microbes Infect. 2019. 10.1016/j.micinf.2018.10.005. 10.1016/j.micinf.2018.10.005 30439507

[42] Nakahira K, Haspel JA, Rathinam VA, Lee SJ, Dolinay T, Lam HC (2011). Autophagy proteins regulate innate immune responses by inhibiting the release of mitochondrial DNA mediated by the NALP3 inflammasome. Nat Immunol.

[43] Shimada K, Crother TR, Karlin J, Dagvadorj J, Chiba N, Chen S (2012). Oxidized mitochondrial DNA activates the NLRP3 inflammasome during apoptosis. Immunity.

[44] Lee GS, Subramanian N, Kim AI, Aksentijevich I, Goldbach-Mansky R, Sacks DB (2012). The calcium-sensing receptor regulates the NLRP3 inflammasome through Ca2+ and cAMP. Nature.

[45] Murakami T, Ockinger J, Yu J, Byles V, McColl A, Hofer AM (2012). Critical role for calcium mobilization in activation of the NLRP3 inflammasome. Proc Natl Acad Sci USA.

[46] Dominic A, Le NT, Takahashi M (2022). Loop between NLRP3 inflammasome and reactive oxygen species. Antioxid Redox Signal.

[47] Muñoz-Planillo R, Kuffa P, Martínez-Colón G, Smith BL, Rajendiran TM, Núñez G (2013). K^+^ efflux is the common trigger of NLRP3 inflammasome activation by bacterial toxins and particulate matter. Immunity.

[48] Christian F, Smith EL, Carmody RJ (2016). The regulation of NF-κB subunits by phosphorylation. Cells.

[49] Park MH, Hong JT (2016). Roles of NF-κB in cancer and inflammatory diseases and their therapeutic approaches. Cells.

[50] Liu T, Zhang L, Joo D, Sun SC (2017). NF-κB signaling in inflammation. Signal Transduct Target Ther.

[51] Zaidi D, Wine E (2018). Regulation of nuclear factor kappa-light-chain-enhancer of activated B cells (NF-κβ) in inflammatory bowel diseases. Front Pediatr.

[52] Schreck R, Rieber P, Baeuerle PA (1991). Reactive oxygen intermediates as apparently widely used messengers in the activation of the NF-kappa B transcription factor and HIV-1. EMBO J.

[53] Chandel NS, Trzyna WC, McClintock DS, Schumacker PT (2000). Role of oxidants in NF-kappa B activation and TNF-alpha gene transcription induced by hypoxia and endotoxin. J Immunol.

[54] Lee YC, Lee KS, Park SJ, Park HS, Lim JS, Park KH (2004). Blockade of airway hyperresponsiveness and inflammation in a murine model of asthma by a prodrug of cysteine, L-2-oxothiazolidine-4-carboxylic acid. FASEB J.

[55] Takada Y, Mukhopadhyay A, Kundu GC, Mahabeleshwar GH, Singh SK, Aggarwal BBJJoBC. Hydrogen peroxide activates NF-κB through tyrosine phosphorylation of IκBα and serine phosphorylation of p65. 2003. 10.1074/jbc.M212389200 12711606

[56] Lee HJ, Hong WG, Woo Y, Ahn JH, Ko HJ, Kim H (2020). Lysophosphatidylcholine enhances bactericidal activity by promoting phagosome maturation via the activation of the NF-κB pathway during salmonella infection in mouse macrophages. Mol Cells.

[57] Storz P, Döppler H, Toker A (2004). Protein kinase Cdelta selectively regulates protein kinase D-dependent activation of NF-kappaB in oxidative stress signaling. Mol Cell Biol.

[58] Song L, Li D, Li X, Ma L, Bai X, Wen Z, et al. Exposure to PM2.5 induces aberrant activation of NF-κB in human airway epithelial cells by downregulating miR-331 expression. Environ Toxicol Pharmacol. 2017. 10.1016/j.etap.2017.02.011. 10.1016/j.etap.2017.02.011 28192748

[59] Chen L, Peng Z, Meng Q, Mongan M, Wang J, Sartor M (2016). Loss of IκB kinase β promotes myofibroblast transformation and senescence through activation of the ROS-TGFβ autocrine loop. Protein Cell.

[60] Li Q, Engelhardt JF (2006). Interleukin-1beta induction of NFkappaB is partially regulated by H2O2-mediated activation of NFkappaB-inducing kinase. J Biol Chem.

[61] Kim EK, Choi EJ (2015). Compromised MAPK signaling in human diseases: an update. Arch Toxicol.

[62] Arthur JS, Ley SC (2013). Mitogen-activated protein kinases in innate immunity. Nat Rev Immunol.

[63] Pelaia C, Vatrella A, Sciacqua A, Terracciano R, Pelaia G (2020). Role of p38-mitogen-activated protein kinase in COPD: pathobiological implications and therapeutic perspectives. Expert Rev Respir Med.

[64] Yeung YT, Bryce NS, Adams S, Braidy N, Konayagi M, McDonald KL (2012). p38 MAPK inhibitors attenuate pro-inflammatory cytokine production and the invasiveness of human U251 glioblastoma cells. J Neurooncol.

[65] Hsieh CC, Papaconstantinou J (2006). Thioredoxin-ASK1 complex levels regulate ROS-mediated p38 MAPK pathway activity in livers of aged and long-lived Snell dwarf mice. FASEB J.

[66] Pan J, Chang Q, Wang X, Son Y, Zhang Z, Chen G (2010). Reactive oxygen species-activated Akt/ASK1/p38 signaling pathway in nickel compound-induced apoptosis in BEAS 2B cells. Chem Res Toxicol.

[67] Schroyer AL, Stimes NW, Abi Saab WF, Chadee DN (2018). MLK3 phosphorylation by ERK1/2 is required for oxidative stress-induced invasion of colorectal cancer cells. Oncogene.

[68] Gonzaga NA, Callera GE, Yogi A, Mecawi AS, Antunes-Rodrigues J, Queiroz RH (2014). Acute ethanol intake induces mitogen-activated protein kinase activation, platelet-derived growth factor receptor phosphorylation, and oxidative stress in resistance arteries. J Physiol Biochem.

[69] Weng MS, Chang JH, Hung WY, Yang YC, Chien MH (2018). The interplay of reactive oxygen species and the epidermal growth factor receptor in tumor progression and drug resistance. J Exp Clin Cancer Res.

[70] Sanchez-Ortiz E, Hahm BK, Armstrong DL, Rossie S (2009). Protein phosphatase 5 protects neurons against amyloid-beta toxicity. J Neurochem.

[71] Xin P, Xu X, Deng C, Liu S, Wang Y, Zhou X (2020). The role of JAK/STAT signaling pathway and its inhibitors in diseases. Int Immunopharmacol.

[72] Morris R, Kershaw NJ, Babon JJ (2018). The molecular details of cytokine signaling via the JAK/STAT pathway. Prot Sci.

[73] Roskoski R (2016). Janus kinase (JAK) inhibitors in the treatment of inflammatory and neoplastic diseases. Pharmacol Res.

[74] Banerjee S, Biehl A, Gadina M, Hasni S, Schwartz DM (2017). JAK-STAT signaling as a target for inflammatory and autoimmune diseases: current and future prospects. Drugs.

[75] Loh CY, Arya A, Naema AF, Wong WF, Sethi G, Looi CY (2019). Signal transducer and activator of transcription (STATs) proteins in cancer and inflammation: functions and therapeutic implication. Front Oncol.

[76] Kaplan MH (2013). STAT signaling in inflammation. JAK-STAT.

[77] Chowdhury FZ, Farrar JD (2013). STAT2: a shape-shifting anti-viral super STAT. Jakstat.

[78] Linher-Melville K, Singh G (2017). The complex roles of STAT3 and STAT5 in maintaining redox balance: Lessons from STAT-mediated xCT expression in cancer cells. Mol Cell Endocrinol.

[79] Shen Z, Jiao K, Teng M, Li Z (2020). Activation of STAT-3 signalling by RECK downregulation via ROS is involved in the 27-hydroxycholesterol-induced invasion in breast cancer cells. Free Radic Res.

[80] Simon AR, Rai U, Fanburg BL, Cochran BH (1998). Activation of the JAK-STAT pathway by reactive oxygen species. Am J Physiol.

[81] Choi S, Lim JW, Kim H (2018). Effect of thiol antioxidants on lipopolysaccharide-induced cyclooxygenase-2 expression in pulmonary epithelial cells. J Physiol Pharmacol.

[82] Liu T, Castro S, Brasier AR, Jamaluddin M, Garofalo RP, Casola A (2004). Reactive oxygen species mediate virus-induced STAT activation: role of tyrosine phosphatases. J Biol Chem.

[83] Motohashi H, Yamamoto M (2004). Nrf2-Keap1 defines a physiologically important stress response mechanism. Trends Mol Med.

[84] Katoh Y, Iida K, Kang MI, Kobayashi A, Mizukami M, Tong KI (2005). Evolutionary conserved N-terminal domain of Nrf2 is essential for the Keap1-mediated degradation of the protein by proteasome. Arch Biochem Biophys.

[85] Katoh Y, Itoh K, Yoshida E, Miyagishi M, Fukamizu A, Yamamoto M (2001). Two domains of Nrf2 cooperatively bind CBP, a CREB binding protein, and synergistically activate transcription. Genes Cells.

[86] Keleku-Lukwete N, Suzuki M, Yamamoto M (2018). An Overview of the advantages of KEAP1-NRF2 system activation during inflammatory disease treatment. Antioxid Redox Signal.

[87] Takaya K, Suzuki T, Motohashi H, Onodera K, Satomi S, Kensler TW (2012). Validation of the multiple sensor mechanism of the Keap1-Nrf2 system. Free Radic Biol Med.

[88] El-Azab MF, Baldowski BR, Mysona BA, Shanab AY, Mohamed IN, Abdelsaid MA (2014). Deletion of thioredoxin-interacting protein preserves retinal neuronal function by preventing inflammation and vascular injury. Br J Pharmacol.

[89] Freigang S, Ampenberger F, Spohn G, Heer S, Shamshiev AT, Kisielow J (2011). Nrf2 is essential for cholesterol crystal-induced inflammasome activation and exacerbation of atherosclerosis. Eur J Immunol.

[90] He Q, You H, Li XM, Liu TH, Wang P, Wang BE (2012). HMGB1 promotes the synthesis of pro-IL-1β and pro-IL-18 by activation of p38 MAPK and NF-κB through receptors for advanced glycation end-products in macrophages. Asian Pac J Cancer Prev.

[91] Hornung V, Bauernfeind F, Halle A, Samstad EO, Kono H, Rock KL (2008). Silica crystals and aluminum salts activate the NALP3 inflammasome through phagosomal destabilization. Nat Immunol.

[92] Lightfield KL, Persson J, Trinidad NJ, Brubaker SW, Kofoed EM, Sauer JD (2011). Differential requirements for NAIP5 in activation of the NLRC4 inflammasome. Infect Immun.

[93] Kong X, Thimmulappa R, Kombairaju P, Biswal S (2010). NADPH oxidase-dependent reactive oxygen species mediate amplified TLR4 signaling and sepsis-induced mortality in Nrf2-deficient mice. J Immunol.

[94] Gao B, Doan A, Hybertson BM (2014). The clinical potential of influencing Nrf2 signaling in degenerative and immunological disorders. Clin Pharmacol Adv Appl.

[95] Zhuang C, Miao Z, Sheng C, Zhang W (2014). Updated research and applications of small molecule inhibitors of Keap1-Nrf2 protein-protein interaction: a review. Curr Med Chem.

[96] Cho HY, Kleeberger SR (2010). Nrf2 protects against airway disorders. Toxicol Appl Pharmacol.

[97] Sarbassov DD, Guertin DA, Ali SM, Sabatini DM (2005). Phosphorylation and regulation of Akt/PKB by the rictor-mTOR complex. Science.

[98] Rosen N, She QB (2006). AKT and cancer–is it all mTOR?. Cancer Cell.

[99] Ma XM, Blenis J (2009). Molecular mechanisms of mTOR-mediated translational control. Nat Rev Mol Cell Biol.

[100] Tokunaga E, Oki E, Egashira A, Sadanaga N, Morita M, Kakeji Y (2008). Deregulation of the Akt pathway in human cancer. Curr Cancer Drug Targets.

[101] Li F, Yao J, Hao Q, Duan Z (2019). Biosci Rep.

[102] Zhang J, Fu Y, Yang P, Liu X, Li Y, Gu ZJAMI. ROS scavenging biopolymers for anti‐inflammatory diseases: classification and formulation. 2020.

[103] Abdel-Mageed HM, Abd El Aziz AE, Abdel Raouf BM, Mohamed SA, Nada D. Antioxidant-biocompatible and stable catalase-based gelatin-alginate hydrogel scaffold with thermal wound healing capability: immobilization and delivery approach. 3 Biotech. 2022. 10.1007/s13205-022-03131-4. 10.1007/s13205-022-03131-4 PMC8859020 35211369

[104] Fu F, Chen Z, Zhao Z, Wang H, Shang L, Gu Z (2017). Bio-inspired self-healing structural color hydrogel. Proc Natl Acad Sci USA.

[105] Wang X, Liu X, Yan X, Zhao P, Ding Y, Xu P (2011). Enzyme-nanoporous gold biocomposite: excellent biocatalyst with improved biocatalytic performance and stability. PLoS ONE.

[106] Zhuang H, Hong Y, Gao J, Chen S, Ma Y, Wang S (2015). A poly(γ-glutamic acid)-based hydrogel loaded with superoxide dismutase for wound healing. J Appl Polym Sci.

[107] Dong Y, Zhuang H, Hao Y, Zhang L, Yang Q, Liu Y (2020). Poly(N-isopropyl-acrylamide)/poly(γ-glutamic acid) thermo-sensitive hydrogels loaded with superoxide dismutase for wound dressing application. Int J Nanomed.

[108] Zhang D, Ren Y, He Y, Chang R, Guo S, Ma S (2022). In situ forming and biocompatible hyaluronic acid hydrogel with reactive oxygen species-scavenging activity to improve traumatic brain injury repair by suppressing oxidative stress and neuroinflammation. Mater Today Biol.

[109] Park C, Park J, Kim WJ, Kim W, Cheong H, Kim SJ (2021). Malonic acid isolated from pinus densiflora inhibits UVB-induced oxidative stress and inflammation in HaCaT keratinocytes. Polymers (Basel).

[110] Qian ZJ, Chen MF, Chen J, Zhang Y, Zhou C, Hong P (2021). Intracellular ethanol-mediated oxidation and apoptosis in HepG2/CYP2E1 cells impaired by two active peptides from seahorse (Hippocampus kuda bleeler) protein hydrolysates via the Nrf2/HO-1 and akt pathways. Food Sci Nutr.

[111] Li J, Li Y, Li Y, Yang Z, Jin H. Physicochemical properties of collagen from acaudina molpadioides and its protective effects against H(2)O(2)-induced injury in RAW264.7 cells. Mar Drugs. 10.3390/md18070370. 10.3390/md18070370 PMC7403972 32708463

[112] Aravinthan A, Park JK, Hossain MA, Sharmila J, Kim HJ, Kang CW, et al. Collagen-based sponge hastens wound healing via decrease of inflammatory cytokines. 3 Biotech. 2018. 10.1007/s13205-018-1497-3. 10.1007/s13205-018-1497-3 PMC6240018 30467532

[113] Jebahi S, Oudadesse H, Jardak N, Khayat I, Keskes H, Khabir A (2013). Biological therapy of strontium-substituted bioglass for soft tissue wound-healing: responses to oxidative stress in ovariectomised rats. Ann Pharm Fr.

[114] Jebahi S, Oudadesse H, Feki He, Rebai T, Keskes H, Pellen P, et al. Antioxidative/oxidative effects of strontium-doped bioactive glass as bone graft. In vivo assays in ovariectomised rats. J Appl Biomed;2012. 10.2478/v10136-012-0009-8.

[115] Li R, Hou X, Li L, Guo J, Jiang W, Shang W (2022). Application of metal-based nanozymes in inflammatory disease: a review. Front Bioeng Biotechnol.

[116] Yao J, Cheng Y, Zhou M, Zhao S, Lin S, Wang X (2018). ROS scavenging Mn(3)O(4) nanozymes for in vivo anti-inflammation. Chem Sci.

[117] Wu Y, Ta HT (2021). Different approaches to synthesising cerium oxide nanoparticles and their corresponding physical characteristics, and ROS scavenging and anti-inflammatory capabilities. J Mater Chem B.

[118] Yu Y, Zhao S, Gu D, Zhu B, Liu H, Wu W (2022). Cerium oxide nanozyme attenuates periodontal bone destruction by inhibiting the ROS-NFκB pathway. Nanoscale.

[119] Niemiec SM, Hilton SA, Wallbank A, Azeltine M, Louiselle AE, Elajaili H, et al. Cerium oxide nanoparticle delivery of microRNA-146a for local treatment of acute lung injury. Nanomed Nanotechnol Biol Med;2021. 10.1016/j.nano.2021.102388. 10.1016/j.nano.2021.102388 PMC7979277 33753282

[120] Dewberry LC, Niemiec SM, Hilton SA, Louiselle AE, Singh S, Sakthivel TS, et al. Cerium oxide nanoparticle conjugation to microRNA-146a mechanism of correction for impaired diabetic wound healing. Nanomedicine : nanotechnology, biology, and medicine. 2022. 10.1016/j.nano.2021.102483. 10.1016/j.nano.2021.102483 PMC9153729 34748956

[121] Huang X, Li L-D, Lyu G-M, Shen B, Han Y, Shi J-L, et al. Chitosan-coated cerium oxide nanocubes accelerate cutaneous wound healing by curtailing persistent inflammation. 2018.

[122] Kyosseva S, Seal S, McGinnis J (2018). Cerium oxide nanoparticles inhibit map kinases activation in the retina of Vldlr mouse model of age-related macular degeneration. Comptes rendus de l'Académie des sciences La vie des sciences.

[123] Kim JW, Mahapatra C, Hong JY, Kim MS, Leong KW, Kim HW (2017). Functional recovery of contused spinal cord in rat with the injection of optimal-dosed cerium oxide nanoparticles. Adv Sci (Weinh).

[124] Peloi KE, Contreras Lancheros CA, Nakamura CV, Singh S, Neal C, Sakthivel TS (2020). Antioxidative photochemoprotector effects of cerium oxide nanoparticles on UVB irradiated fibroblast cells. Colloids Surf B.

[125] Ribeiro FM, de Oliveira MM, Singh S, Sakthivel TS, Neal CJ, Seal S (2020). Ceria nanoparticles decrease UVA-induced fibroblast death through cell redox regulation leading to cell survival, migration and proliferation. Front Bioeng Biotechnol.

[126] Peloi KE, Ratti BA, Nakamura CV, Neal CJ, Sakthivel TS, Singh S (2021). Engineered nanoceria modulate neutrophil oxidative response to low doses of UV-B radiation through the inhibition of reactive oxygen species production. J Biomed Mater Res Part A.

[127] Soh M, Kang DW, Jeong HG, Kim D, Kim DY, Yang W (2017). Ceria-zirconia nanoparticles as an enhanced multi-antioxidant for sepsis treatment. Angew Chem Int Ed Engl.

[128] Miriyala S, Spasojevic I, Tovmasyan A, Salvemini D, Vujaskovic Z, St Clair D (2012). Manganese superoxide dismutase, MnSOD and its mimics. Biochim Biophys Acta.

[129] Xiong Y, Zhang Y, Zhou C, Yu T (2023). ROS scavenging Manganese-loaded mesoporous silica nanozymes for catalytic anti-inflammatory therapy. Adv Powder Technol.

[130] Ai Y, You J, Gao J, Wang J, Sun H-b, Ding M, et al. Multi-shell nanocomposites based multienzyme mimetics for efficient intracellular antioxidation. Nano Res;2021. 10.1007/s12274-020-3267-x.

[131] Hu S, Wang L, Li J, Li D, Zeng H, Chen T (2023). Catechol-modified and MnO(2)-nanozyme-reinforced hydrogel with improved antioxidant and antibacterial capacity for periodontitis treatment. ACS Biomater Sci Eng.

[132] Liu T, Xiao B, Xiang F, Tan J, Chen Z, Zhang X (2020). Ultrasmall copper-based nanoparticles for reactive oxygen species scavenging and alleviation of inflammation related diseases. Nat Commun.

[133] Peng Y, He D, Ge X, Lu Y, Chai Y, Zhang Y, et al. Construction of heparin-based hydrogel incorporated with Cu5.4O ultrasmall nanozymes for wound healing and inflammation inhibition. Bioact Mater. 2021. 10.1016/j.bioactmat.2021.02.006. 10.1016/j.bioactmat.2021.02.006 PMC7960791 33778192

[134] Zhang S, Chen J, Lian M-L, Yang W-S, Chen X (2022). An engineered, self-propelled nanozyme as reactive oxygen species scavenger. Chem Eng J.

[135] Chen Y, Wang Y, Chen Z, Cai J, Li K, Huang H (2022). NIR-driven polydopamine-based nanoenzymes as ROS scavengers to suppress osteoarthritis progression. Mater Today Nano.

[136] Yoshihisa Y, Honda A, Zhao QL, Makino T, Abe R, Matsui K (2010). Protective effects of platinum nanoparticles against UV-light-induced epidermal inflammation. Exp Dermatol.

[137] Hou W, Ye C, Chen M, Gao W, Xie X, Wu J (2021). Excavating bioactivities of nanozyme to remodel microenvironment for protecting chondrocytes and delaying osteoarthritis. Bioact Mater.

[138] Huang Y, Xu Q, Zhang J, Yin Y, Pan Y, Zheng Y (2022). Prussian blue scavenger ameliorates hepatic ischemia-reperfusion injury by inhibiting inflammation and reducing oxidative stress. Front Immunol.

[139] Sahu A, Jeon J, Lee MS, Yang HS, Tae G (2021). Antioxidant and anti-inflammatory activities of Prussian blue nanozyme promotes full-thickness skin wound healing. Mater Sci Eng C Mater Biol Appl.

[140] Dong C, Ma X, Huang Y, Zhang Y, Gao X (2022). Carbon dots nanozyme for anti-inflammatory therapy via scavenging intracellular reactive oxygen species. Front Bioeng Biotechnol.

[141] Ma Y, Gao W, Zhang Y, Yang M, Yan X, Zhang Y (2022). Biomimetic MOF nanoparticles delivery of C-dot nanozyme and CRISPR/Cas9 system for site-specific treatment of ulcerative colitis. ACS Appl Mater Interfaces.

[142] Tu Z, Zhong Y, Hu H, Shao D, Haag R, Schirner M (2022). Design of therapeutic biomaterials to control inflammation. Nat Rev Mater.

[143] Doleski PH, Ten Caten MV, Passos DF, Castilhos LG, Leal DBR, Machado VS (2017). Toxoplasmosis treatment with diphenyl diselenide in infected mice modulates the activity of purinergic enzymes and reduces inflammation in spleen. Exp Parasitol.

[144] Zhang C, Wang H, Liang W, Yang Y, Cong C, Wang Y (2021). Diphenyl diselenide protects motor neurons through inhibition of microglia-mediated inflammatory injury in amyotrophic lateral sclerosis. Pharmacol Res.

[145] Brüning CA, Prigol M, Luchese C, Jesse CR, Duarte MM, Roman SS (2012). Protective effect of diphenyl diselenide on ischemia and reperfusion-induced cerebral injury: involvement of oxidative stress and pro-inflammatory cytokines. Neurochem Res.

[146] Xu L, Gong C, Li G, Wei J, Wang T, Meng W (2018). Ebselen suppresses inflammation induced by Helicobacter pylori lipopolysaccharide via the p38 mitogen-activated protein kinase signaling pathway. Mol Med Rep.

[147] Chen D, Zheng R, Su J, Lai J, Chen H, Ning Z (2022). Inhibition of H1N1 influenza virus-induced apoptosis by Ebselen through ROS-mediated ATM/ATR signaling pathways. Biol Trace Elem Res.

[148] Tewari R, Sharma V, Koul N, Ghosh A, Joseph C, Hossain Sk U (2009). Ebselen abrogates TNFalpha induced pro-inflammatory response in glioblastoma. Mol Oncol.

[149] Huang X, Liu X, Luo Q, Liu J, Shen J (2011). Artificial selenoenzymes: designed and redesigned. Chem Soc Rev.

[150] Sands KN, Tuck TA, Back TG (2018). Cyclic seleninate esters, spirodioxyselenuranes and related compounds: new classes of biological antioxidants that emulate glutathione peroxidase. Chemistry.

[151] Pu HL, Chiang WL, Maiti B, Liao ZX, Ho YC, Shim MS (2014). Nanoparticles with dual responses to oxidative stress and reduced ph for drug release and anti-inflammatory applications. ACS Nano.

[152] Nimse SB, Pal DJRA. Free radicals, natural antioxidants, and their reaction mechanisms. 2015.

[153] Zhang ZY, Jiang M, Fang J, Yang MF, Zhang S, Yin YX (2017). Enhanced therapeutic potential of nano-curcumin against subarachnoid hemorrhage-induced blood-brain barrier disruption through inhibition of inflammatory response and oxidative stress. Mol Neurobiol.

[154] Doggui S, Sahni JK, Arseneault M, Dao L, Ramassamy C (2012). Neuronal uptake and neuroprotective effect of curcumin-loaded PLGA nanoparticles on the human SK-N-SH cell line. J Alzheimers Dis.

[155] Fernandes M, Lopes I, Magalhães L, Sárria MP, Machado R, Sousa JC (2021). Novel concept of exosome-like liposomes for the treatment of Alzheimer's disease. J Control Release.

[156] Qian F, Han Y, Han Z, Zhang D, Zhang L, Zhao G (2021). In Situ implantable, post-trauma microenvironment-responsive, ROS depletion hydrogels for the treatment of traumatic brain injury. Biomaterials.

[157] Chen K, Pan H, Ji D, Li Y, Duan H, Pan W (2021). Curcumin-loaded sandwich-like nanofibrous membrane prepared by electrospinning technology as wound dressing for accelerate wound healing. Mater Sci Eng C Mater Biol Appl.

[158] Hu B, Gao M, Boakye-Yiadom KO, Ho W, Yu W, Xu X (2021). An intrinsically bioactive hydrogel with on-demand drug release behaviors for diabetic wound healing. Bioact Mater.

[159] Zhang X, Feng J, Feng W, Xu B, Zhang K, Ma G (2022). Glycosaminoglycan-based hydrogel delivery system regulates the wound microenvironment to rescue chronic wound healing. ACS Appl Mater Interfaces.

[160] Teng S, Joseph MJ, Yu H, Hu C, Li X, Hu C (2022). A narrative review of the protective effects of curcumin in treating ischemia-reperfusion injury. Ann Transl Med.

[161] Du B, Zhao M, Wang Y, Yu L, Jiao Q, Bai Y (2022). Folic acid-targeted pluronic F127 micelles improve oxidative stress and inhibit fibrosis for increasing AKI efficacy. Eur J Pharmacol.

[162] Yuan R, Li Y, Han S, Chen X, Chen J, He J (2022). Fe-curcumin nanozyme-mediated reactive oxygen species scavenging and anti-inflammation for acute lung injury. ACS Cent Sci.

[163] Hu J, Yang L, Yang P, Jiang S, Liu X, Li Y (2020). Polydopamine free radical scavengers. Biomater Sci.

[164] Liu H, Qu X, Tan H, Song J, Lei M, Kim E (2019). Role of polydopamine's redox-activity on its pro-oxidant, radical-scavenging, and antimicrobial activities. Acta Biomater.

[165] Guo Y, Baschieri A, Mollica F, Valgimigli L, Cedrowski J, Litwinienko G, et al. Hydrogen atom transfer from HOO(.) to ortho-quinones explains the antioxidant activity of polydopamine. Angew Chem Int Ed Engl. 2021. 10.1002/anie.202101033. 10.1002/anie.202101033 PMC8362028 33876878

[166] Zheng B, Deng G, Zheng J, Li Y, Wang B, Ding X (2022). Self-polymerized polydopamine-based nanoparticles for acute kidney injury treatment through inhibiting oxidative damages and inflammatory. Int J Biochem Cell Biol.

[167] Zhao H, Zeng Z, Liu L, Chen J, Zhou H, Huang L (2018). Polydopamine nanoparticles for the treatment of acute inflammation-induced injury. Nanoscale.

[168] Bao X, Zhao J, Sun J, Hu M, Yang X (2018). Polydopamine nanoparticles as efficient scavengers for reactive oxygen species in periodontal disease. ACS Nano.

[169] Fu Y, Zhang J, Wang Y, Li J, Bao J, Xu X (2021). Reduced polydopamine nanoparticles incorporated oxidized dextran/chitosan hybrid hydrogels with enhanced antioxidative and antibacterial properties for accelerated wound healing. Carbohydr Polym.

[170] Battaglini M, Marino A, Carmignani A, Tapeinos C, Cauda V, Ancona A (2020). Polydopamine nanoparticles as an organic and biodegradable multitasking tool for neuroprotection and remote neuronal stimulation. ACS Appl Mater Interfaces.

[171] Yan M, Zhu L, Wu S, Cao Y, Mou N, Chi Q (2022). ROS responsive polydopamine nanoparticles to relieve oxidative stress and inflammation for ameliorating acute inflammatory bowel. Biomater Adv.

[172] Sahiner N, Sagbas S, Aktas N, Silan C (2016). Inherently antioxidant and antimicrobial tannic acid release from poly(tannic acid) nanoparticles with controllable degradability. Colloids Surf B Biointerfaces.

[173] Ni Z, Yu H, Wang L, Huang Y, Lu H, Zhou H (2022). Multistage ROS-responsive and natural polyphenol-driven prodrug hydrogels for diabetic wound healing. ACS Appl Mater Interfaces.

[174] Li Y, Fu R, Duan Z, Zhu C, Fan D (2022). Construction of multifunctional hydrogel based on the tannic acid-metal coating decorated MoS(2) dual nanozyme for bacteria-infected wound healing. Bioact Mater.

[175] Shi W, Kong Y, Su Y, Kuss MA, Jiang X, Li X (2021). Tannic acid-inspired, self-healing, and dual stimuli responsive dynamic hydrogel with potent antibacterial and anti-oxidative properties. J Mater Chem B.

[176] Pan W, Qi X, Xiang Y, You S, Cai E, Gao T (2022). Facile formation of injectable quaternized chitosan/tannic acid hydrogels with antibacterial and ROS scavenging capabilities for diabetic wound healing. Int J Biol Macromol.

[177] Li R, Fan Y, Liu L, Ma H, Gong D, Miao Z (2022). Ultrathin hafnium disulfide atomic crystals with ROS-scavenging and colon-targeting capabilities for inflammatory bowel disease treatment. ACS Nano.

[178] Yang X, Yang J, Ye Z, Zhang G, Nie W, Cheng H, et al. Physiologically inspired mucin coated *Escherichia coli* Nissle 1917 enhances biotherapy by regulating the pathological microenvironment to improve intestinal colonization. ACS Nano. 2022. 10.1021/acsnano.1c09681. 10.1021/acsnano.1c09681 35230097

[179] Li M, Liu P, Xue Y, Liang Y, Shi J, Han X (2020). Tannic acid attenuates hepatic oxidative stress, apoptosis and inflammation by activating the Keap1-Nrf2/ARE signaling pathway in arsenic trioxide-toxicated rats. Oncol Rep.

[180] Duan R, Sun K, Fang F, Wang N, He R, Gao Y (2022). An ischemia-homing bioengineered nano-scavenger for specifically alleviating multiple pathogeneses in ischemic stroke. J Nanobiotechnology.

[181] Ullah R, Ali G, Baseer A, Irum Khan S, Akram M, Khan S (2022). Tannic acid inhibits lipopolysaccharide-induced cognitive impairment in adult mice by targeting multiple pathological features. Int Immunopharmacol.

[182] Son HY, Koo BI, Lee JB, Kim KR, Kim W, Jang J (2018). Tannin-titanium oxide multilayer as a photochemically suppressed ultraviolet filter. ACS Appl Mater Interfaces.

[183] Byun H, Jang GN, Hong MH, Yeo J, Shin H, Kim WJ (2022). Biomimetic anti-inflammatory and osteogenic nanoparticles self-assembled with mineral ions and tannic acid for tissue engineering. Nano Converg.

[184] Lee JY, Lim H, Ahn JW, Jang D, Lee SH, Park K (2018). Design of a 3D BMP-2-delivering tannylated pcl scaffold and its anti-oxidant, anti-inflammatory, and osteogenic effects in vitro. Int J Mol Sci.

[185] Choi S, Jo HS, Song H, Kim HJ, Oh JK, Cho JW (2021). Multifunctional tannic acid-alendronate nanocomplexes with antioxidant, anti-inflammatory, and osteogenic potency. Nanomaterials (Basel).

[186] Li Y, Chen M, Yan J, Zhou W, Gao S, Liu S (2021). Tannic acid/Sr(2+)-coated silk/graphene oxide-based meniscus scaffold with anti-inflammatory and anti-ROS functions for cartilage protection and delaying osteoarthritis. Acta Biomater.

[187] Kim SA, Jong YC, Kang MS, Yu CJ (2022). Antioxidation activity of molecular hydrogen via protoheme catalysis in vivo: an insight from ab initio calculations. J Mol Model.

[188] Ohta S (2014). Molecular hydrogen as a preventive and therapeutic medical gas: initiation, development and potential of hydrogen medicine. Pharmacol Ther.

[189] Tian Y, Zhang Y, Wang Y, Chen Y, Fan W, Zhou J (2021). Hydrogen, a novel therapeutic molecule, regulates oxidative stress, inflammation, and apoptosis. Front Physiol.

[190] Yang M, Dong Y, He Q, Zhu P, Zhuang Q, Shen J (2020). Hydrogen: a novel option in human disease treatment. Oxid Med Cell Longev.

[191] You IS, Sharma S, Fadriquela A, Bajgai J, Thi TT, Rahman MH (2021). Antioxidant properties of hydrogen gas attenuates oxidative stress in airway epithelial cells. Molecules.

[192] Saitoh Y, Yonekura N, Matsuoka D, Matsumoto A (2022). Molecular hydrogen suppresses Porphyromonas gingivalis lipopolysaccharide-induced increases in interleukin-1 alpha and interleukin-6 secretion in human gingival cells. Mol Cell Biochem.

[193] Yin H, Feng Y, Duan Y, Ma S, Guo Z, Wei Y (2022). Hydrogen gas alleviates lipopolysaccharide-induced acute lung injury and inflammatory response in mice. J Inflamm (Lond).

[194] Hirano SI, Ichikawa Y, Sato B, Yamamoto H, Takefuji Y, Satoh F (2021). Potential therapeutic applications of hydrogen in chronic inflammatory diseases: possible inhibiting role on mitochondrial stress. Int J Mol Sci.

[195] Ishibashi T, Ichikawa M, Sato B, Shibata S, Hara Y, Naritomi Y (2015). Improvement of psoriasis-associated arthritis and skin lesions by treatment with molecular hydrogen: a report of three cases. Mol Med Rep.

[196] Wan WL, Lin YJ, Chen HL, Huang CC, Shih PC, Bow YR (2017). In situ nanoreactor for photosynthesizing H(2) gas to mitigate oxidative stress in tissue inflammation. J Am Chem Soc.

[197] Wan WL, Tian B, Lin YJ, Korupalli C, Lu MY, Cui Q (2020). Photosynthesis-inspired H(2) generation using a chlorophyll-loaded liposomal nanoplatform to detect and scavenge excess ROS. Nat Commun.

[198] Criado-Gonzalez M, Mecerreyes D (2022). Thioether-based ROS responsive polymers for biomedical applications. J Mater Chem B.

[199] Hasan AA, Kalinina E, Tatarskiy V, Shtil A (2022). The thioredoxin system of mammalian cells and its modulators. Biomedicines.

[200] Matsuzawa A (2017). Thioredoxin and redox signaling: Roles of the thioredoxin system in control of cell fate. Arch Biochem Biophys.

[201] Scott KA, Njardarson JT (2018). Analysis of US FDA-approved drugs containing sulfur atoms. Top Curr Chem (Cham).

[202] Zhu D, Chen W, Lin W, Li Y, Liu X (2021). Reactive oxygen species-responsive nanoplatforms for nucleic acid-based gene therapy of cancer and inflammatory diseases. Biomed Mater.

[203] Rajkovic O, Gourmel C, d'Arcy R, Wong R, Rajkovic I, Tirelli N, et al. Reactive oxygen species‐responsive nanoparticles for the treatment of ischemic stroke. 2019.

[204] Yoo D, Magsam AW, Kelly AM, Stayton PS, Kievit FM, Convertine AJ (2017). Core-cross-linked nanoparticles reduce neuroinflammation and improve outcome in a mouse model of traumatic brain injury. ACS Nano.

[205] O'Grady KP, Kavanaugh TE, Cho H, Ye H, Gupta MK, Madonna MC (2018). Drug-free ROS sponge polymeric microspheres reduce tissue damage from ischemic and mechanical injury. ACS Biomater Sci Eng.

[206] Liu S, Zhang Q, Yu J, Shao N, Lu H, Guo J (2020). Absorbable thioether grafted hyaluronic acid nanofibrous hydrogel for synergistic modulation of inflammation microenvironment to accelerate chronic diabetic wound healing. Adv Healthc Mater.

[207] Elisia I, Nakamura H, Lam V, Hofs E, Cederberg R, Cait J (2016). DMSO represses inflammatory cytokine production from human blood cells and reduces autoimmune arthritis. PLoS ONE.

[208] de Abreu Costa L, Henrique Fernandes Ottoni M, Dos Santos MG, Meireles AB, Gomes de Almeida V, de Fátima Pereira W, et al. Dimethyl Sulfoxide (DMSO) Decreases cell proliferation and TNF-α, IFN-γ, and IL-2 cytokines production in cultures of peripheral blood lymphocytes. Molecules. 2017. 10.3390/molecules22111789. 10.3390/molecules22111789 PMC6150313 29125561

[209] Chen Q, Olashaw N, Wu J (1995). Participation of reactive oxygen species in the lysophosphatidic acid-stimulated mitogen-activated protein kinase kinase activation pathway. J Biol Chem.

[210] Murphy B, Bhattacharya R, Mukherjee P (2019). Hydrogen sulfide signaling in mitochondria and disease. FASEB J.

[211] Wedmann R, Bertlein S, Macinkovic I, Böltz S, Miljkovic J, Muñoz LE (2014). Working with "H2S": facts and apparent artifacts. Nitric Oxide.

[212] Filipovic MR, Miljkovic J, Allgäuer A, Chaurio R, Shubina T, Herrmann M (2012). Biochemical insight into physiological effects of H_2_S: reaction with peroxynitrite and formation of a new nitric oxide donor, sulfinyl nitrite. Biochem J.

[213] Kida K, Ichinose F (2015). Hydrogen sulfide and neuroinflammation. Handb Exp Pharmacol.

[214] Stocker R, Yamamoto Y, McDonagh AF, Glazer AN, Ames BN (1987). Bilirubin is an antioxidant of possible physiological importance. Science.

[215] Liu Y, Liu J, Tetzlaff W, Paty DW, Cynader MS (2006). Biliverdin reductase, a major physiologic cytoprotectant, suppresses experimental autoimmune encephalomyelitis. Free Radic Biol Med.

[216] Huang Y, Li J, Li W, Ai N, Jin H (2022). Biliverdin/bilirubin redox pair protects lens epithelial cells against oxidative stress in age-related cataract by regulating NF-κB/iNOS and Nrf2/HO-1 pathways. Oxid Med Cell Longev.

[217] Saha S, Buttari B, Panieri E, Profumo E, Saso L (2020). An overview of Nrf2 signaling pathway and its role in inflammation. Molecules.

[218] Kim DE, Lee Y, Kim M, Lee S, Jon S, Lee S-H (2017). Bilirubin nanoparticles ameliorate allergic lung inflammation in a mouse model of asthma. Biomaterials.

[219] Yao Q, Jiang X, Zhai YY, Luo LZ, Xu HL, Xiao J (2020). Protective effects and mechanisms of bilirubin nanomedicine against acute pancreatitis. J Control Release.

[220] Xue S, Zhou X, Sang W, Wang C, Lu H, Xu Y (2021). Cartilage-targeting peptide-modified dual-drug delivery nanoplatform with NIR laser response for osteoarthritis therapy. Bioactive Materials.

[221] Lee Y, Sugihara K, Gillilland MG, Jon S, Kamada N, Moon JJ (2020). Hyaluronic acid–bilirubin nanomedicine for targeted modulation of dysregulated intestinal barrier, microbiome and immune responses in colitis. Nat Mater.

[222] Lee Y, Kim H, Kang S, Lee J, Park J, Jon S (2016). Bilirubin nanoparticles as a nanomedicine for anti-inflammation therapy. Angew Chem Int Ed.

[223] Chen Z, Chen Y, Hao W, Shui M, Zhang J, Zhou H (2023). Oral delivery of transformable bilirubin self-assembled system for targeted therapy of colitis. Adv Healthc Mater.

[224] Ai W, Bae S, Ke Q, Su S, Li R, Chen Y (2021). Bilirubin nanoparticles protect against cardiac ischemia/reperfusion injury in mice. J Am Heart Assoc.

[225] Kim JY, Lee DY, Kang S, Miao W, Kim H, Lee Y (2017). Bilirubin nanoparticle preconditioning protects against hepatic ischemia-reperfusion injury. Biomaterials.

[226] Keum H, Kim TW, Kim Y, Seo C, Son Y, Kim J (2020). Bilirubin nanomedicine alleviates psoriatic skin inflammation by reducing oxidative stress and suppressing pathogenic signaling. J Control Release.

[227] Jiang X, Yao Q, Xia X, Tang Y, Sun M, Li Y (2022). Self-assembled nanoparticles with bilirubin/JPH203 alleviate imiquimod-induced psoriasis by reducing oxidative stress and suppressing Th17 expansion. Chem Eng J.

[228] Keum H, Kim D, Kim J, Kim TW, Whang C-H, Jung W (2021). A bilirubin-derived nanomedicine attenuates the pathological cascade of pulmonary fibrosis. Biomaterials.

[229] Barbieri SS, Cavalca V, Eligini S, Brambilla M, Caiani A, Tremoli E (2004). Apocynin prevents cyclooxygenase 2 expression in human monocytes through NADPH oxidase and glutathione redox-dependent mechanisms. Free Radic Biol Med.

[230] Kim SY, Moon KA, Jo HY, Jeong S, Seon SH, Jung E (2012). Anti-inflammatory effects of apocynin, an inhibitor of NADPH oxidase, in airway inflammation. Immunol Cell Biol.

[231] Qin YY, Li M, Feng X, Wang J, Cao L, Shen XK (2017). Combined NADPH and the NOX inhibitor apocynin provides greater anti-inflammatory and neuroprotective effects in a mouse model of stroke. Free Radical Biol Med.

[232] Kim SK, Rho SJ, Kim SH, Kim SY, Song SH, Yoo JY (2019). Protective effects of diphenyleneiodonium, an NADPH oxidase inhibitor, on lipopolysaccharide-induced acute lung injury. Clin Exp Pharmacol Physiol.

[233] Mehmood A, Zhao L, Wang C, Hossen I, Nadeem M (2020). Stevia residue extract alone and combination with allopurinol attenuate hyperuricemia in fructose-PO-induced hyperuricemic mice. J Food Biochem.

[234] Wan L, Chen G, Jian S, Yin XJ, Zhu H (2018). Antioxidant and xanthine oxidase inhibitory properties and LC-MS/MS identification of compoundsof ethanolic extract from Mulberry leaves. Acta Sci Pol Technol Aliment.

[235] Wu L, Zhou C, Wu J, Chen S, Tian Z, Du Q (2020). Corticosterone inhibits LPS-induced NLRP3 inflammasome priming in macrophages by suppressing xanthine oxidase. Mediators Inflamm.

[236] Cerqua I, Musella S, Peltner LK, D'Avino D, Di Sarno V, Granato E, et al. Discovery and optimization of indoline-based compounds as dual 5-LOX/seh inhibitors: in vitro and in vivo anti-inflammatory characterization. J Med Chem. 2022. 10.1021/acs.jmedchem.2c00817. 10.1021/acs.jmedchem.2c00817 PMC9661480 36318728

[237] Nagesh Khadri MJ, Khamees HA, Kouser S, Zabiulla, Khanum SA. Synthesis, analgesic, anti-inflammatory, ulcerogenic evaluation, and docking study of (benzoylphenoxy)-N-{5-[2-methylphenyl-6-chlorobenzoxazole]} acetamides as COX/5-LOX inhibitor. J Mol Struct. 2023. 10.1016/j.molstruc.2022.134240.

[238] Mavuso S, Choonara YE, Marimuthu T, Kumar P, du Toit LC, Kondiah PPD (2016). A dual pH/Redox responsive copper-ligand nanoliposome bioactive complex for the treatment of chronic inflammation. Int J Pharm.

[239] Li YR, Zhu H (2021). Nanoceria potently reduce superoxide fluxes from mitochondrial electron transport chain and plasma membrane NADPH oxidase in human macrophages. Mol Cell Biochem.

[240] Stahr PL, Grewal R, Eckert GP, Keck CM (2021). Investigating hesperetin nanocrystals with tailor-made sizes for the prevention and treatment of Alzheimer's disease. Drug Deliv Transl Res.

[241] Babylon L, Grewal R, Stahr PL, Eckert RW, Keck CM, Eckert GP (2021). Hesperetin nanocrystals improve mitochondrial function in a cell model of early Alzheimer disease. Antioxidants (Basel).

[242] Mijiritsky E, Gardin C, Ferroni L, Lacza Z, Zavan B (2020). Albumin-impregnated bone granules modulate the interactions between mesenchymal stem cells and monocytes under in vitro inflammatory conditions. Mater Sci Eng C Mater Biol Appl.

[243] Fokam D, Hoskin D (2020). Instrumental role for reactive oxygen species in the inflammatory response. Front Biosci (Landmark Ed).

[244] Pelletier M, Lepow TS, Billingham LK, Murphy MP, Siegel RM (2012). New tricks from an old dog: mitochondrial redox signaling in cellular inflammation. Semin Immunol.

[245] Huang X, He D, Pan Z, Luo G, Deng J (2021). Reactive-oxygen-species-scavenging nanomaterials for resolving inflammation. Mater Today Biol.

[246] Hou W, Ye C, Chen M, Gao W, Xie X, Wu J, et al. Excavating bioactivities of nanozyme to remodel microenvironment for protecting chondrocytes and delaying osteoarthritis. 2021. 10.1016/j.bioactmat.2021.01.016 PMC7848724 33553826

[247] Huang Y, Xu Q, Zhang J, Yin Y, Pan Y, Zheng Y, et al. Prussian blue scavenger ameliorates hepatic ischemia-reperfusion injury by inhibiting inflammation and reducing oxidative stress. 2022. 10.3389/fimmu.2022.891351 PMC9174572 35693813

[248] Sahu A, Jeon J, Lee MS, Yang HS, Tae GJMs, engineering. C Mfba. Antioxidant and anti-inflammatory activities of Prussian blue nanozyme promotes full-thickness skin wound healing. 2020. 10.1016/j.msec.2020.111596 33321640

[249] Dong C, Ma X, Huang Y, Zhang Y, Gao XJFiB, Biotechnology. Carbon dots nanozyme for anti-inflammatory therapy via scavenging intracellular reactive oxygen species. 2022. 10.3389/fbioe.2022.943399 PMC9420844 36046669

[250] Ma Y, Gao W, Zhang Y, Yang M, Yan X, Zhang Y, et al. Biomimetic MOF nanoparticles delivery of C-dot nanozyme and CRISPR/cas9 system for site-specific treatment of ulcerative colitis. 2022. 10.1021/acsami.1c21700 35099925

[251] Da J, Li Y, Zhang K, Ren J, Wang J, Liu X (2022). Functionalized prussian blue nanozyme as dual-responsive drug therapeutic nanoplatform against maxillofacial infection via macrophage polarization. Int J Nanomed.

[252] Chen X, Li C, Cao X, Jia X, Chen X, Wang Z (2022). Mitochondria-targeted supramolecular coordination container encapsulated with exogenous itaconate for synergistic therapy of joint inflammation. Theranostics.

[253] Sacco P, Decleva E, Tentor F, Menegazzi R, Borgogna M, Paoletti S (2017). Butyrate-loaded chitosan/hyaluronan nanoparticles: a suitable tool for sustained inhibition of ROS release by activated neutrophils. Macromol Biosci.

[254] Selvaraj V, Manne ND, Arvapalli R, Rice KM, Nandyala G, Fankenhanel E, et al. Effect of cerium oxide nanoparticles on sepsis induced mortality and NF-κB signaling in cultured macrophages. Nanomedicine (London, England). 2015. 10.2217/nnm.14.205. 10.2217/nnm.14.205 25955124

